# Dephosphorylation of astrocyte elevated gene-1 protein upregulates eIF4E expression to promote gastric cancer progression

**DOI:** 10.1016/j.jbc.2025.110687

**Published:** 2025-09-04

**Authors:** Li Zhao, Xixi Qian, Yaoyao Fan, Huiling Li, Zuhao Zhang, Chen Chen, Lulu Yang, Shaorong Yu, Xuerong Wang, Wenbin Huang

**Affiliations:** 1 Department of Pharmacology, School of Basic Medical Sciences, Nanjing Medical University, Nanjing, Jiangsu Province, China; 2 Department of Pathology, the First Affiliated Hospital of Henan University of Science & Technology, Luoyang, Henan province, China; 3 Department of Oncology, the Affiliated Cancer Hospital of Nanjing Medical University & Jiangsu Cancer Hospital & Jiangsu Institute of Cancer Research, Nanjing, Jiangsu Province, China; 4 Department of Pathology, Nanjing First Hospital, Nanjing Medical University, Nanjing, Jiangsu province, China; 5 The College of Basic Medicine and Forensic Medicine, Henan University of Science & Technology, Luoyang, Henan province, China

**Keywords:** gastric cancer, phosphorylation, astrocyte elevated gene-1, eukaryotic translation initiation factor 4E, phosphotase protein 1 regulatory subunit 21, NF-κB

## Abstract

Protein phosphorylation modification plays an important role in regulating protein activity. Astrocyte elevated gene-1 (AEG-1), an adaptor protein, promotes the progression of various types of cancers by protein–protein interactions. We previously demonstrated that AEG-1 promoted the growth and metastasis of gastric cancer by upregulating the expression of oncogenic eukaryotic translation initiation factor 4E. However, a role for AEG-1 phosphorylation is not known. In this study, we identified phosphorylation of serines 426 (S426) and 308 (S308) as the inactive status of AEG-1. Decreased AEG-1 S426 phosphorylation in human gastric cancer tissues was correlated with the cancer progression, whereas AEG-1 S426 dephosphorylation upregulated eukaryotic translation initiation factor 4E transcription expression. This dephosphorylation was facilitated by AEG-1 S308 dephosphorylation through a mechanism involving the activation of p65 NF-κB. Double phosphorylation of AEG-1 S308–426 significantly inhibited the growth and migration of cancer cells while also suppressing the growth and metastasis of gastric cancer in nude mouse models. Mechanistically, the phosphatase protein 1 regulatory subunit 21 bound to AEG-1 and mediated the protein phosphatase 1 catalytic subunit to induce the loss of AEG-1 phosphorylation. Our findings highlight a role for AEG-1 dephosphorylation in the progression of gastric cancer and provide potential new targets for cancer therapy.

Gastric cancer is the fifth most commonly diagnosed cancer worldwide, with an incidence of 4.9% among all cancer types, accounting for over 968,000 new global cases in 2022. It is also the fifth leading cause of cancer death, with a mortality rate of 6.8% and approximately 660,000 deaths globally in 2022 (1). The main causes of patient death are metastasis and recurrence. A frequent metastatic site is the peritoneum, and dissemination can occur synchronously with primary tumors or after curative surgery, contributing to the low survival rate of gastric cancer patients (2). Some patients have greatly benefited from human epidermal growth factor receptor 2–targeted therapy and immunotherapy; however, cytotoxic chemotherapy remains the primary therapeutic strategy, especially for patients with advanced-stage disease (3). Therefore, identifying key molecules and clarifying the mechanisms that drive gastric cancer progression are urgently needed to provide potential new targets and drugs for cancer therapy.

One potential target is astrocyte elevated gene-1 (AEG-1), also known as metadherin (MTDH) or lysine-rich CEACAM1 coisolated protein (LYRIC). The AEG-1 gene was initially cloned from primary human fetal astrocytes after induction by HIV infection or tumor necrosis factor alpha (TNF-α) (4). In the past 2 decades, increased expression of AEG-1 has been reported to correlate with poor prognosis and to promote the progression of various types of cancer, including breast cancer, gastric cancer, and malignant gliomas (5). AEG-1 can activate pivotal protumor signaling pathways, such as PI3K/Akt/mTOR, MAPK/ERK, and NF-κB, as well as upregulate the expression of tumorigenic molecules, such as c-Myc, MMP9, and eukaryotic translation initiation factor 4E (eIF4E) (6). Mechanistically, AEG-1 is an adaptor protein, mainly functioning through interactions with other proteins, such as SND1 (staphylococcal nuclease and Tudor domain–containing 1), NF-κB, and BCCIP (BRCA2 interacting protein), thereby influencing downstream signaling pathways (7, 8, 9, 10). The binding of AEG-1 to SND1 has been shown to augment breast cancer progression, including tumor growth, metastasis, drug resistance, and suppression of the tumor microenvironment (11). The discovery that targeting the AEG-1–SND1 interaction using a small molecule significantly suppresses breast cancer progression provides a potential new therapeutic approach (12, 13).

Our previous work revealed a correlation between elevated AEG-1 expression and the progression and poor prognosis of gastric cancer (14). AEG-1 promoted the growth and metastasis of gastric cancer by upregulating the expression of eIF4E (15, 16). However, the upstream signals that regulate AEG-1 modification in gastric cancer have not been elucidated, especially AEG-1 interaction proteins that are largely unknown.

Protein phosphorylation at serine, threonine, and tyrosine residues regulates protein function by modifying kinase activity, spatial conformation, subcellular localization, protein–protein interactions, and the binding affinity for small-molecule drugs. In addition, phosphorylation frequently triggers further post-translational modifications, such as ubiquitination and palmitoylation (17). Protein phosphorylation is a dynamic and reversible process, orchestrated by protein kinases, which transfer phosphate groups from ATP to substrate proteins, and protein phosphatases (PPs), which catalyze the removal of these phosphate groups (18). Although kinases and phosphatases share roles in phosphate transfer, their catalytic mechanisms are quite different. Kinases directly recognize substrates and phosphorylate specific amino acid residues using ATP. Specific kinase inhibitors are well developed and have great benefit to cancer patients. However, serine/threonine phosphatases, particularly those in the phosphoprotein phosphatase (PPP) family, generally function as multiprotein complexes composed of catalytic and regulatory subunits (19). Representative catalytic subunits of the PPP family include PP1, PP2A, PP2B, PP4, PP5, and PP6 (20). Regulatory subunits play crucial roles in defining substrate specificity (21, 22). Identifying specific regulatory subunits of phosphatases has emerged as a promising strategy for developing allosteric drugs in targeted cancer therapy.

One potential target is the AEG-1 protein, which contains many serine, threonine, and tyrosine residues that could serve as potential phosphorylation sites based on structural predictions. Quantitative phosphoproteomic analyses have revealed multiple phosphorylation sites, such as T143, S298, serine 426 (S426), and S568, on AEG-1 following mitotic stimulation or stem cell differentiation (23, 24, 25). To date, one study has confirmed that phosphorylation of AEG-1 at serine 298 (S298) is catalyzed by inhibitory kappa B kinase beta following TNF-α treatment and that the phosphorylated protein subsequently activates the NF-κB signaling pathway to promote cell proliferation (26). Whether other potential phosphorylation sites on AEG-1 affect its structure, protein–protein interactions, or biological functions remains largely unknown.

In the present study, we conducted immunoprecipitation (IP) and mass spectrometry (MS) analyses of AEG-1 to identify its phosphorylation sites following stimulation by CXC chemokine ligand 12 (CXCL12), which modulates metastasis, particularly the peritoneal dissemination of gastric cancer (27, 28). We found that the loss of phosphorylation at S426 and serine 308 (S308) on AEG-1 promoted the growth and metastasis of gastric cancer through upregulation of eIF4E and the p65 NF-κB signaling pathways. Mechanistically, protein phosphatase 1 regulatory subunit 21 (PPP1R21) binds to AEG-1 and mediates dephosphorylation by the PP1 catalytic subunit (PP1cs) in gastric cancer cells. Notably, we developed an antibody that specifically detects phosphorylation of AEG-1 at S426, and we found that this phosphorylation in human gastric cancer tissues decreased with disease progression. Our findings highlight the role of AEG-1 phosphorylation in gastric cancer and suggest potential therapeutic targets.

## Results

### Phosphorylation of AEG-1 at S426 was decreased during the progression of gastric cancer

**Figure 1:**
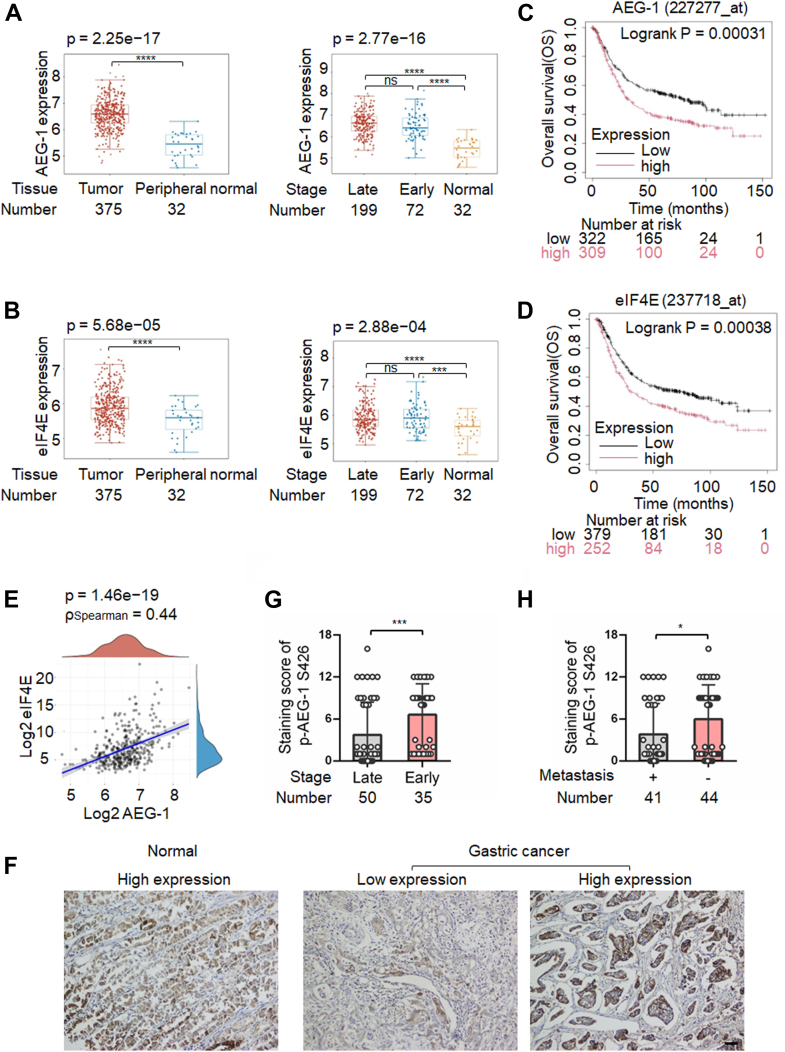
**Phosphorylation levels of AEG-1 at S426 decreased, whereas total protein levels of AEG-1 and eIF4E increased in human gastric cancer tissues as the disease progressed**. *A*, *B*, and *E*, bioinformatic analysis of AEG-1 expression, eIF4E expression, and their correlation in gastric cancer tissues based on TCGA datasets using the Home for Researchers platform (https://www.home-for-researchers.com/). Late stage: stage Ⅱb, Ⅲ, and Ⅳ; early stage: stage Ⅰb, stage Ⅱa. *C*–*D*, correlation between AEG-1 or eIF4E expression and patient survival based on TCGA datasets, analyzed using the Kaplan–Meier Plotter tool (http://kmplot.com/analysis). *F*, representative IHC images showing p-AEG-1 S426 expression in normal and gastric cancer tissues. The scale bar represents 200 μm. *G*–*H*, quantification of p-AEG-1 S426 IHC staining in gastric cancer tissues from 85 patients. Points, staining score of individual patient; columns, means of all patient values; the bars represent SD. ∗*p* < 0.05, ∗∗*p* < 0.01, and ∗∗∗*p* < 0.001. AEG-1, astrocyte elevated gene-1; eIF4E, eukaryotic translation initiation factor 4E; IHC, immunohistochemistry; S426, serine 426; TCGA, The Cancer Genome Atlas. AEG-1, astrocyte elevated gene-1; CXCL12, CXC chemokine ligand 12; eIF4E, eukaryotic translation initiation factor 4E; PP1, protein phosphatase 1; PPP1R21, protein phosphatase 1 regulatory subunit 21; S308, serine 308; S426, serine 426; SRB, sulforhodamine B; TCGA, The Cancer Genome Atlas; TNF-α, tumor necrosis factor alpha.

We previously reported that AEG-1 promoted the growth and metastasis of gastric cancer by upregulating eIF4E expression levels (15, 29). Moreover, AEG-1 expression was higher in human gastric cancer tissues than in normal gastric mucosal tissues and was even more elevated in late-stage cancer tissues compared with early stage ones (14). Although eIF4E exhibited similar expression patterns in gastric cancer (16), we had not yet examined whether AEG-1 and eIF4E expression levels were correlated in human cancer tissues or whether their expression was associated with patient survival. To address this, we analyzed AEG-1 and eIF4E expression using the Home for Researchers platform (https://www.home-for-researchers.com) based on The Cancer Genome Atlas datasets. We found that AEG-1 expression was higher in gastric cancer tissues than in peripheral normal tissues and was even further elevated in late-stage cancers compared with in early stage ones (Fig. 1*A*). eIF4E showed a similar expression trend (Fig. 1*B*). Higher expression of either AEG-1 or eIF4E was associated with poorer patient survival (Fig. 1, *C* and *D*) using the Kaplan–Meier Plotter tool (http://kmplot.com/analysis). Importantly, AEG-1 expression positively correlated with eIF4E expression, supporting our previous finding that AEG-1 enhances eIF4E expression to promote gastric cancer progression (Fig. 1*E*) (15, 29).

Given that online databases predict multiple phosphorylation sites in AEG-1, we next investigated whether AEG-1 is phosphorylated and whether this modification affects its function. We used CXCL12 to stimulate gastric cancer cells, as it is a key chemokine that drives metastasis, particularly peritoneal dissemination, in gastric cancer (30). Gastric cancer cell line BGC823 was treated with CXCL12 for 1 h followed by AEG-1 IP. The immunoprecipitates were then subjected to MS analysis to identify phosphorylation sites. Two phosphorylation sites on AEG-1—S426 and T492—were detected, with a slight decrease in S426 phosphorylation observed following CXCL12 treatment (Supplementary File S1). Based on literature analysis, S426 is consistently identified as the most prominent phosphorylation site, often exhibiting the highest Mascot Score across multiple published MS datasets, particularly in mitotic cells, whereas T492 phosphorylation is rarely reported (23, 24, 25, 31, 32). Thus, despite the identification of both S426 and T492 phosphorylation sites, we prioritized S426 for further investigation as supported by previous literature.

We then generated a monoclonal antibody specific to phosphorylated AEG-1 at S426 (p-AEG-1 S426) to examine its phosphorylation levels in cell lines and human tissues. The antibody’s specificity was validated *via* dot blot using phosphorylated and nonphosphorylated peptides (Fig. S1*A*). Moreover, CXCL12 reduced p-AEG-1 S426 levels in SGC7901 cells, confirming the MS data by Western blotting (Fig. S1*B*). Furthermore, we constructed two mutant AEG-1 expression plasmids: a nonphosphorylatable mutant (S426A; serine to alanine) and a phosphomimetic mutant (S426D; serine to aspartate), using the WT AEG-1 plasmid as a template. Western blotting showed that p-AEG-1 S426 levels were higher in S426D-overexpressing cells than in WT or S426A-expressing cells (Fig. S1*C*). These results confirm the antibody’s specificity and functionality *in vitro* and *in vivo*.

Using this antibody, we assessed endogenous p-AEG-1 S426 expression in multiple gastric cancer cell lines (BGC823, MGC803, and MKN45) and in normal gastric epithelial GES-1 cells (Fig. S1*D*). Immunohistochemistry (IHC) revealed detectable phosphorylation of AEG-1 at S426 in both gastric cancer and normal tissues (Fig. 1*F*). In a cohort of 85 gastric cancer patients, tumors from patients with advanced pTNM stage exhibited lower p-AEG-1 S426 expression than those at early stages (Fig. 1*G*). Similarly, tumors from patients with lymph node metastasis had lower p-AEG-1 S426 levels than those without metastasis (Fig. 1*H*).

These results indicate that CXCL12 induces dephosphorylation of AEG-1 at S426, and decreased p-AEG-1 S426 levels are associated with gastric cancer progression. We hypothesize that dephosphorylation represents the active form of AEG-1 that contributes to cancer development.

### Dephosphorylation of AEG-1 at S426 upregulated eIF4E transcription and promoted the growth and migration of gastric cancer cells

**Figure 2:**
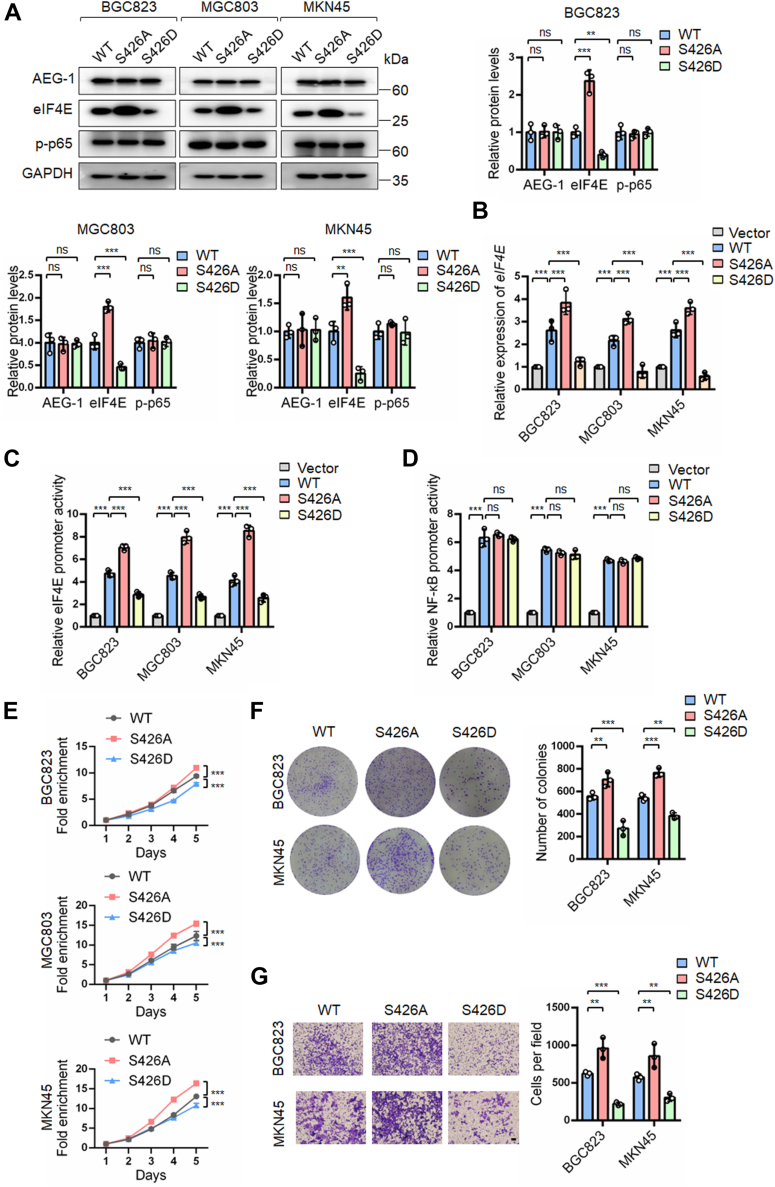
**Dephosphorylation of AEG-1 S426 upregulated eIF4E expression and promoted the growth and migration of gastric cancer cells**. *A*–*B*, gastric cancer cells were transfected with the indicated AEG-1 plasmids for 48 h and analyzed by Western blotting and qRT–PCR. Western blot bands were quantified by densitometry, and values were normalized to GAPDH. Data are presented as mean ± SD from three independent experiments. *C*–*D*, cells were cotransfected with AEG-1 plasmids and luciferase reporter plasmids of eIF4E promoter or NF-κB activity and subjected to dual-luciferase reporter assay. Columns, means of three replicate determinations. *E*–*G*, cells transfected with AEG-1 plasmids were subjected to SRB assay, colony formation assay, and transwell migration assay. Points, means of four replicate determinations (SRB); columns, means of three replicate determinations (colony formation); columns, means of three microscopic fields (transwell); the bars represent SD. ∗*p* < 0.05, ∗∗*p* < 0.01, and ∗∗∗*p* < 0.001. The scale bar represents 200 μm. AEG-1, astrocyte elevated gene-1; eIF4E, eukaryotic translation initiation factor 4E; qRT–PCR, quantitative PCR; S426, serine 426; SRB, sulforhodamine B.

To determine the function of AEG-1 S426 phosphorylation, we overexpressed AEG-1 WT, S426A, and S426D constructs in gastric cancer cells and examined their effects. The expression levels of eIF4E were used as a readout for AEG-1 activity, based on our previous findings that eIF4E upregulation mediates the oncogenic function of AEG-1 in gastric cancer (15). Overexpression of AEG-1 S426D in BGC823 gastric cancer cells significantly reduced eIF4E protein levels, whereas AEG-1 S426A overexpression increased eIF4E levels compared with AEG-1 WT (Fig. 2*A*). We then explored how AEG-1 regulates eIF4E expression. Using quantitative RT–PCR (qRT–PCR) and a dual-luciferase reporter assay, we found that AEG-1 WT overexpression increased eIF4E mRNA levels and promoter activity. In contrast, AEG-1 S426D overexpression significantly decreased both eIF4E mRNA expression and promoter activity, whereas AEG-1 S426A overexpression enhanced them, compared with AEG-1 WT (Fig. 2, *B* and *C*). These findings indicate that phosphorylation of AEG-1 at S426 inhibits eIF4E transcription.

To further investigate the underlying mechanism, we performed bioinformatics analysis using the JASPAR platform (https://jaspar.elixir.no/) and the Human Transcription Factor Database (http://bioinfo.life.hust.edu.cn/HumanTFDB/#!/), which identified NF-κB as a potential transcription factor regulating the eIF4E promoter (Fig. S2). Given previous reports that AEG-1 physically binds to and activates p65 NF-κB, we assessed NF-κB transcriptional activity in response to different AEG-1 phosphorylation states using a luciferase reporter plasmid containing three tandem NF-κB binding sites (8, 9). As expected, AEG-1 WT increased NF-κB transcriptional activity. However, neither AEG-1 S426A nor S426D altered NF-κB activity compared with AEG-1 WT (Fig. 2*D*). Similarly, the phosphorylation levels of p65 NF-κB (p-p65 NF-κB) remained unchanged upon overexpression of AEG-1 S426A or S426D (Fig. 2*A*). These results suggest that while dephosphorylation of AEG-1 at S426 enhances eIF4E transcription, this effect is likely independent of direct p65 NF-κB activation.

We then examined the biological function of AEG-1 in gastric cancer cells. Compared with AEG-1 WT, overexpression of AEG-1 S426D suppressed, whereas S426A enhanced, the growth and migration of gastric cancer cells, as determined by sulforhodamine B (SRB) assay, colony formation assay, and transwell migration assay (Fig. 2, *E*–*G*). It indicates that phosphorylation of AEG-1 at S426 is the inactive status of AEG-1, whereas dephosphorylation is the active status.

These findings suggest that dephosphorylation of AEG-1 at S426 activates the eIF4E promoter, thereby upregulating eIF4E transcription and promoting the growth and migration of gastric cancer cells.

### eIF4E transcription induced by AEG-1 S426 dephosphorylation was facilitated by AEG-1 S308 dephosphorylation through activation of NF-κB

The observation that AEG-1 S426 phosphorylation did not affect p65 NF-κB activity prompted us to explore additional AEG-1 dephosphorylation sites that might activate p65 NF-κB and thereby enhance eIF4E transcription induced by AEG-1 S426 dephosphorylation. Krishnan *et al*. (26) previously reported that TNF-α-induced phosphorylation of AEG-1 at S298 activated p65 NF-κB; however, other phosphorylation sites, such as S308 and S426, were not investigated. Given that we were looking for a dephosphorylation site induced by TNF-α, we focused on S308, which was located near S298.

**Figure 3:**
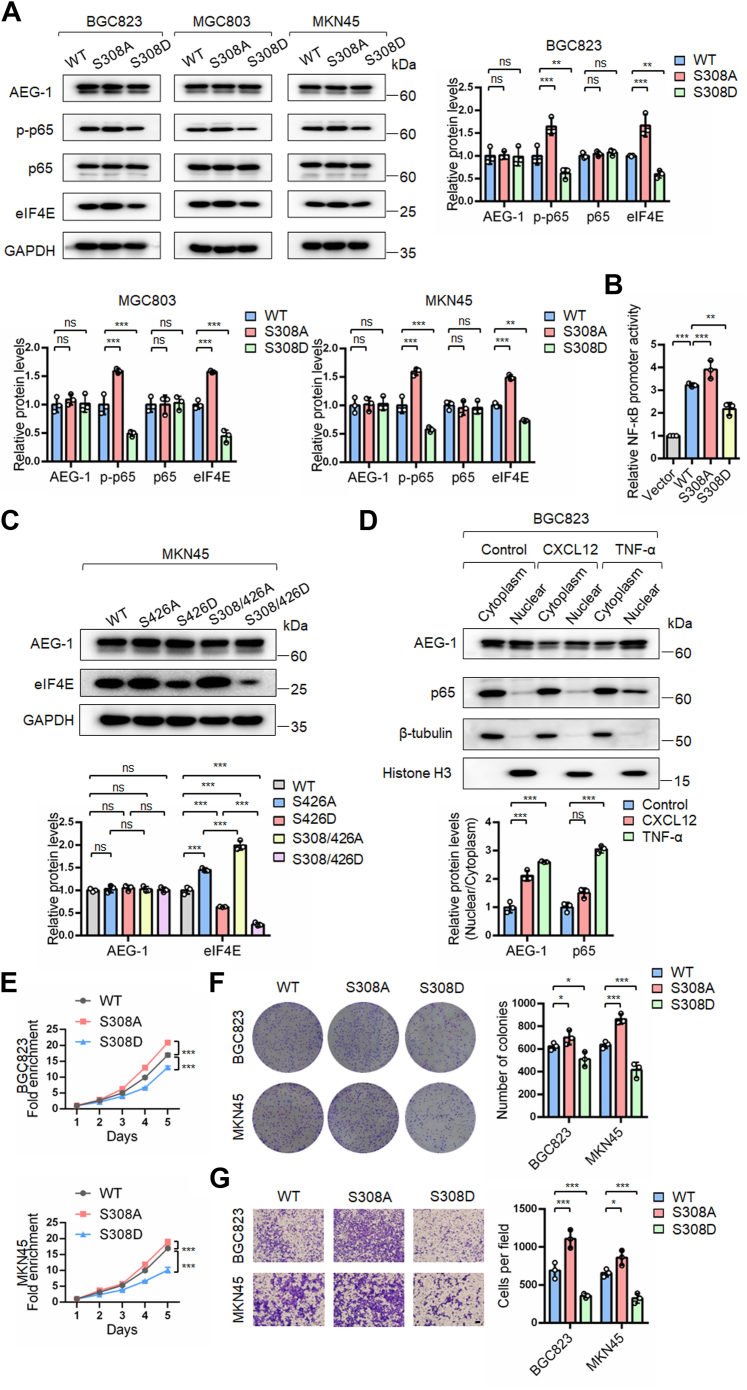
**Dephosphorylation of AEG-1 at S308 facilitated eIF4E upregulation induced by S426 dephosphorylation, activated NF-**κ**B signaling, and promoted the growth and migration of gastric cancer cells**. *A*–*C*, gastric cancer cells were transfected with the indicated plasmids for 48 h and analyzed by Western blotting and dual-luciferase reporter assays. Western blot bands were quantified by densitometry, and values were normalized to GAPDH. Data are presented as mean ± SD from three independent experiments. *D*, BGC823 cells were treated with CXCL12 or TNF-α for 24 h. Cytoplasmic and nuclear fractions were extracted and analyzed by Western blotting. Western blot bands were quantified by densitometry. Cytoplasmic and nuclear protein levels were normalized to β-tubulin and histone H3, respectively. The ratio of nuclear to cytoplasmic protein was calculated and presented as mean ± SD from three independent experiments. *E*–*G*, cells transfected with AEG-1 plasmids were subjected to SRB assay, colony formation assay, and transwell migration assay. Points, means of four replicate determinations (SRB); columns, means of three replicate determinations (colony formation); columns, means of three microscopic fields (transwell); the bars represent SD. ∗*p* < 0.05, ∗∗*p* < 0.01, and ∗∗∗*p* < 0.001. The scale bar represents 200 μm. AEG-1, astrocyte elevated gene-1; CXCL12, CXC chemokine ligand 12; eIF4E, eukaryotic translation initiation factor 4E; S308, serine 308; SRB, sulforhodamine B; TNF-α, tumor necrosis factor alpha.

To investigate this, we generated AEG-1 S308A (nonphosphorylatable) and S308D (phosphomimetic) mutants. Overexpression of AEG-1 S308D significantly inhibited NF-κB signaling, as evidenced by reduced p-p65 NF-κB levels and decreased NF-κB luciferase reporter activity compared with AEG-1 WT, whereas S308A showed only a modest enhancement (Fig. 3, *A* and *B*). Notably, AEG-1 S308D overexpression reduced eIF4E protein levels, whereas S308A increased eIF4E levels, but the effect was less pronounced than that of S426 mutants, indicating a facilitated effect of S308 dephosphorylation in AEG-1-activated eIF4E transcription (Fig. 3*A*).

We speculated that dual phosphorylation at S308 and S426 might further suppress eIF4E transcription. To test this, we constructed dual mutant AEG-1 plasmids: S308/426A and S308/426D. The S308/426D double mutant exhibited a stronger suppressive effect on eIF4E expression than the S426D single mutant (Fig. 3*C*). In parallel, we observed that TNF-α treatment induced nuclear translocation of both AEG-1 and p65 NF-κB, whereas CXCL12 only promoted nuclear translocation of AEG-1 (Fig. 3*D*). These observations support our hypothesis that AEG-1 S308 dephosphorylation activates NF-κB, which in turn facilitates eIF4E transcription induced by AEG-1 S426 dephosphorylation. To assess the functional consequences of S308 phosphorylation, we examined cell growth and migration using the SRB assay, colony formation assay, and transwell assay. AEG-1 S308D overexpression suppressed cell growth and migration, whereas S308A overexpression enhanced these processes compared with AEG-1 WT (Fig. 3, *E*–*G*).

These findings suggest that dephosphorylation of AEG-1 S308 activates p65 NF-κB and enhances eIF4E transcriptional activation induced by AEG-1 S426 dephosphorylation.

### Dual dephosphorylation of AEG-1 at S308 and S426 promoted the growth and migration of gastric cancer cells

**Figure 4:**
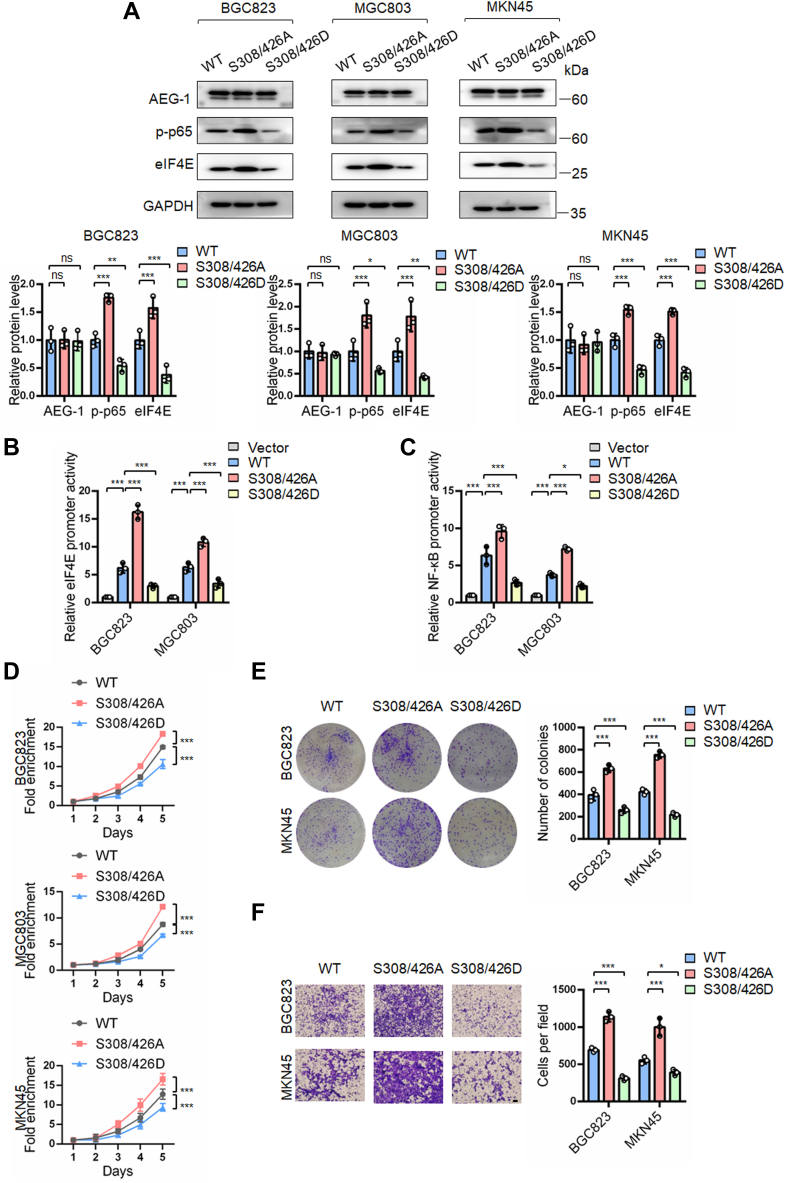
**Dual phosphorylation of AEG-1 at S308/S426 downregulated eIF4E expression and NF-κB signaling and suppressed the growth and migration of gastric cancer cells.***A*–*C*, gastric cancer cells were transfected with the indicated plasmids for 48 h and analyzed by Western blotting and dual-luciferase reporter assays. Western blot bands were quantified by densitometry, and values were normalized to GAPDH. Data are presented as mean ± SD from three independent experiments. *D*–*F*, cells were subjected to SRB assay, colony formation assay, and transwell migration assay. Points, means of four replicate determinations (SRB); columns, means of three replicate determinations (colony formation); columns, means of three microscopic fields (transwell); the bars represent SD. ∗*p* < 0.05, ∗∗*p* < 0.01, and ∗∗∗*p* < 0.001. The scale bar represents 200 μm. AEG-1, astrocyte elevated gene-1; eIF4E, eukaryotic translation initiation factor 4E; S308, serine 308; S426, serine 426; SRB, sulforhodamine B.

Given that AEG-1 integrates various extracellular signals beyond CXCL12 and TNF-α to promote the growth of gastric cancer cells, we evaluated the AEG-1 S308/426 double mutant under standard serum-containing culture conditions. The AEG-1 S308/426A mutant significantly upregulated eIF4E expression and promoter activity (Fig. 4, *A* and *B*), accompanied by increased p-p65 NF-κB levels and transcriptional activity (Fig. 4, *A* and *C*), as determined by Western blotting and luciferase reporter assays. Furthermore, the AEG-1 S308/426A mutant enhanced cell growth and migration, whereas the S308/426D mutant suppressed these processes compared with AEG-1 WT (Fig. 4, *D*–*F*).

To further validate these findings, we established BGC823 cell lines stably overexpressing AEG-1 WT, S308/426D, or vector control using lentiviral transduction. Cells expressing the AEG-1 S308/426D mutant displayed reduced levels of eIF4E and p-p65 NF-κB, along with decreased growth and migration capabilities compared with WT-expressing cells (Fig. S3).

These results suggest that dephosphorylation of AEG-1 at both S308 and S426 enhances p65 NF-κB activity and eIF4E expression, thereby promoting the growth and migration of gastric cancer cells.

### Dual phosphorylation of AEG-1 at S308 and S426 inhibited the growth and metastasis of gastric cancer in nude mouse models

**Figure 5:**
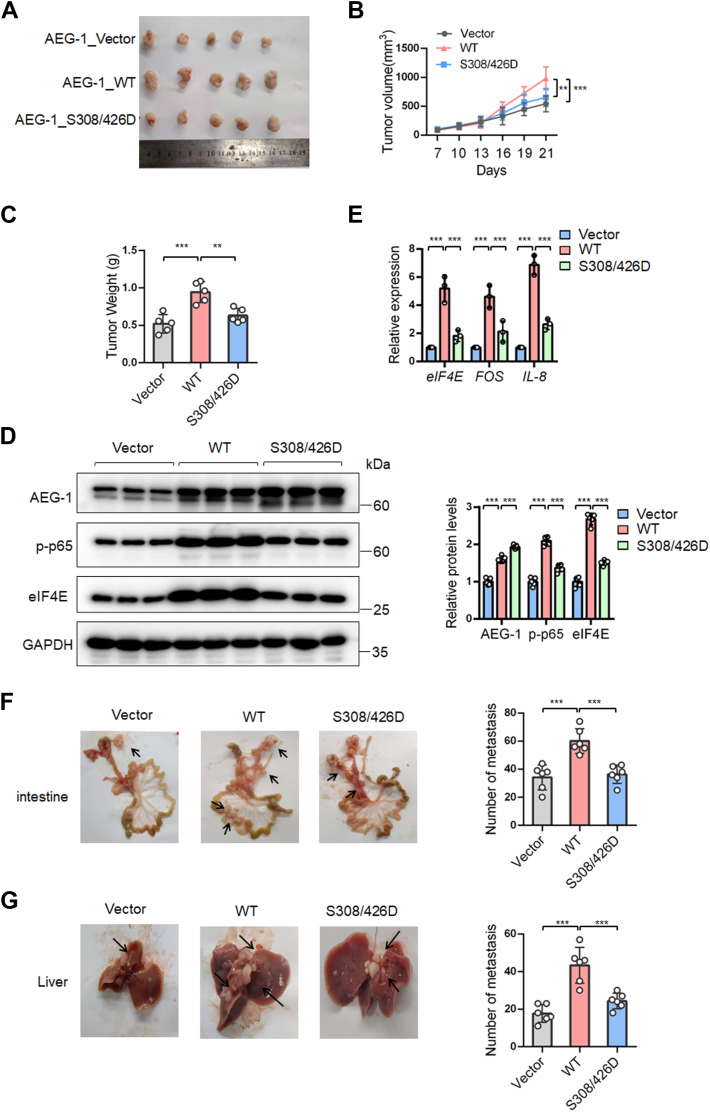
**Dual phosphorylation of AEG-1 at S308/426 suppressed eIF4E expression and NF-κB signaling and inhibited the growth and migration of gastric cancer in nude mouse models.***A*–*C*, tumor photos, tumor size, and tumor weight from nude mice subcutaneously injected with BGC823 stable cell lines overexpressing AEG-1 WT, S308/426D mutant, or vector control. *D*–*E*, Western blot and qRT–PCR analysis of tumor samples. Representative Western blot images from three mice per group are shown (Western blot images from the other two mice in each group are shown in Fig. S4). Western blot bands were quantified by densitometry, and values were normalized to GAPDH. Data are presented as mean ± SD from five mice per group. *F*–*G*, representative photos and quantification of metastatic lesions on the intestines and liver surfaces of nude mice. Points, means of five mice per group in *B*; points, each mouse in *C*, *F*, and *G*; columns, means of five mice per group in *E*; the bars represent SD. ∗*p* < 0.05, ∗∗*p* < 0.01, and ∗∗∗*p* < 0.001. AEG-1, astrocyte elevated gene-1; eIF4E, eukaryotic translation initiation factor 4E; qRT–PCR, quantitative RT–PCR; S308, serine 308; S426, serine 426.

We also examined the effects of AEG-1 S308/426 phosphorylation on tumor growth in a nude mouse xenograft model. BGC823 cells stably overexpressing AEG-1 WT, the S308/426D mutant, or vector control were subcutaneously inoculated into nude mice. Compared with the vector group, AEG-1 WT expression significantly promoted tumor growth, as evidenced by increased tumor volume and weight. However, expression of the AEG-1 S308/426D mutant significantly suppressed tumor growth compared with WT (Fig. 5, *A*–*C*). Tumor tissues from mice bearing AEG-1 S308/426D–expressing cells exhibited lower protein levels of eIF4E and p-p65 NF-κB compared with the WT group (Figs. 5*D* and S4). The mRNA levels of eIF4E and the NF-κB downstream targets *IL-8* and *FOS* were significantly reduced in tumors from the S308/426D group compared with WT (Fig. 5*E*).

To assess the impact of AEG-1 S308/426 phosphorylation on metastasis, we used a peritoneal dissemination model by injecting cancer cells into the abdominal cavity of nude mice, mimicking clinical metastasis patterns in gastric cancer. One month after injection, small metastatic nodules were observed on the surfaces of the small intestine, liver, and peritoneum. Mice bearing AEG-1 S308/426D–expressing cells developed fewer metastatic lesions on the small intestine and liver compared with the WT group (Fig. 5, *F* and *G*).

These findings suggest that the AEG-1 S308/426 dual phosphorylation represents the inactive state of AEG-1, which downregulates eIF4E and NF-κB signaling pathways, thereby suppressing the growth and metastasis of gastric cancer.

### PPP1R21 interacted with AEG-1 to mediate its dephosphorylation

**Figure 6:**
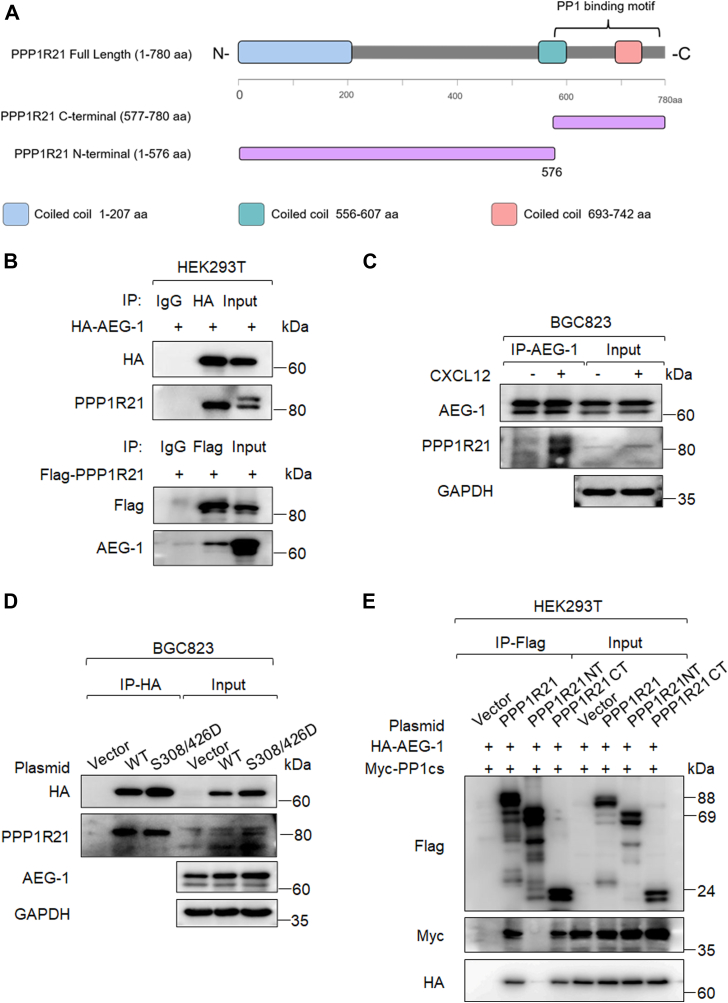
**PPP1R21 induced AEG-1 dephosphorylation.***A*, schematic diagrams of full-length and truncated PPP1R21 constructs. *B*, HEK293T cells were transfected with HA-AEG-1 or FLAG-PPP1R21 plasmids for 48 h. Whole-cell lysates were prepared and subjected to coimmunoprecipitation (co-IP) using anti-HA or anti-FLAG antibodies, as indicated. *C*, BGC823 cells were treated with 100 ng/ml CXCL12 for 24 h. Whole-cell lysates were prepared and subjected to co-IP using the anti-AEG-1 antibody. *D*, BGC823 cells stably expressing AEG-1 WT or the phosphorylated S308/426D mutant (with HA tag) were cultured for 24 h. Whole-cell lysates were prepared and subjected to co-IP using the anti-HA antibody. *E*, HEK293T cells were transfected with full-length or truncated FLAG-PPP1R21 plasmids, as described in *A*, along with Myc-PP1cs and HA-AEG-1 plasmids for 48 h. Whole-cell lysates were subjected to co-IP using the anti-FLAG antibody. Coimmunoprecipitated proteins from *B* to *E* were analyzed by Western blotting. aa, amino acid; AEG-1, astrocyte elevated gene-1; CXCL12, CXC chemokine ligand 12; HEK293T, human embryonic kidney 293T cell line; PP1cs, PP1 catalytic subunit; PPP1R21, protein phosphatase 1 regulatory subunit 21.

We further investigated the upstream mechanisms responsible for AEG-1 dephosphorylation, with particular emphasis on phosphatase-mediated processes. Interestingly, a separate ongoing project in our laboratory focusing on PPP1R21 identified AEG-1 (also known as Protein LYRIC, MTDH) as a binding partner. In this study, full-length PPP1R21 with a FLAG tag (FLAG-PPP1R21 FL) was overexpressed in human embryonic kidney 293T (HEK293T) cells (Fig. 6*A*), followed by co-IP using an anti-FLAG antibody or control immunoglobulin G (IgG). The resulting precipitates were subjected to MS analysis. Importantly, AEG-1 was identified as an interacting protein in this dataset (Supplementary File S2). Subsequently, structural prediction analysis suggested that PPP1R21 may function as a regulatory subunit of PP1. Moreover, previous proteomic studies demonstrated a physical interaction between PPP1R21 and PP1 through peptide-level analyses (33). Based on these findings, we hypothesized that PPP1R21 functions as a regulatory subunit that mediates PP1-catalyzed dephosphorylation of AEG-1.

Then, we developed a specific antibody recognizing PPP1R21. Western blotting showed that both anti-FLAG and anti-PPP1R21 antibodies detected a band of the expected molecular weight (∼88 kDa) in BGC823 cells transfected with the plasmid (Fig. S5*A*). Knockdown of PPP1R21 by siRNA markedly reduced this signal, confirming the specificity of the antibody (Fig. S5*B*).

We then proceeded to confirm the interaction between PPP1R21 and AEG-1 using co-IP assays in HEK293T cells cotransfected with HA-AEG-1 and FLAG-PPP1R21 constructs (Fig. 6*A*). IP with HA pulled down endogenous PPP1R21, whereas FLAG IP pulled down endogenous AEG-1 (Fig. 6*B*). Moreover, CXCL12 promoted the binding of endogenous PPP1R21 to AEG-1 in gastric cancer cells (Fig. 6*C*). Notably, the AEG-1 S308/426D mutant exhibited reduced interaction with PPP1R21 compared with WT, indicating that PPP1R21 binds dephosphorylated AEG-1 (Fig. 6*D*). To map the interaction domains, we constructed N-terminal (FLAG-PPP1R21 NT) and C-terminal (FLAG-PPP1R21 CT) truncations of PPP1R21 (Fig. 6*A*). It should be noted that the C-terminal truncation contained the motif (577–769 amino acids) that interacted with PP1cs as previously reported (33). Then HEK293T cells were cotransfected with Myc-PP1cs and HA-AEG-1 plasmids, along with FLAG-tagged full-length or truncated PPP1R21 constructs. PPP1R21 was immunoprecipitated using an anti-FLAG antibody, followed by immunoblotting with anti-Myc and anti-HA antibodies to detect PP1cs and AEG-1, respectively. The results showed that both AEG-1 and PP1cs predominantly interact with the C-terminal region as well as the full-length form of PPP1R21 (Fig. 6*E*). These findings suggest that CXCL12 promotes the dephosphorylation of AEG-1 through PPP1R21-mediated recruitment of PP1cs to AEG-1.

### PPP1R21 promoted AEG-1 dephosphorylation and enhanced the growth and migration of gastric cancer cells, and its expression was associated with poor prognosis in gastric cancer patients

**Figure 7:**
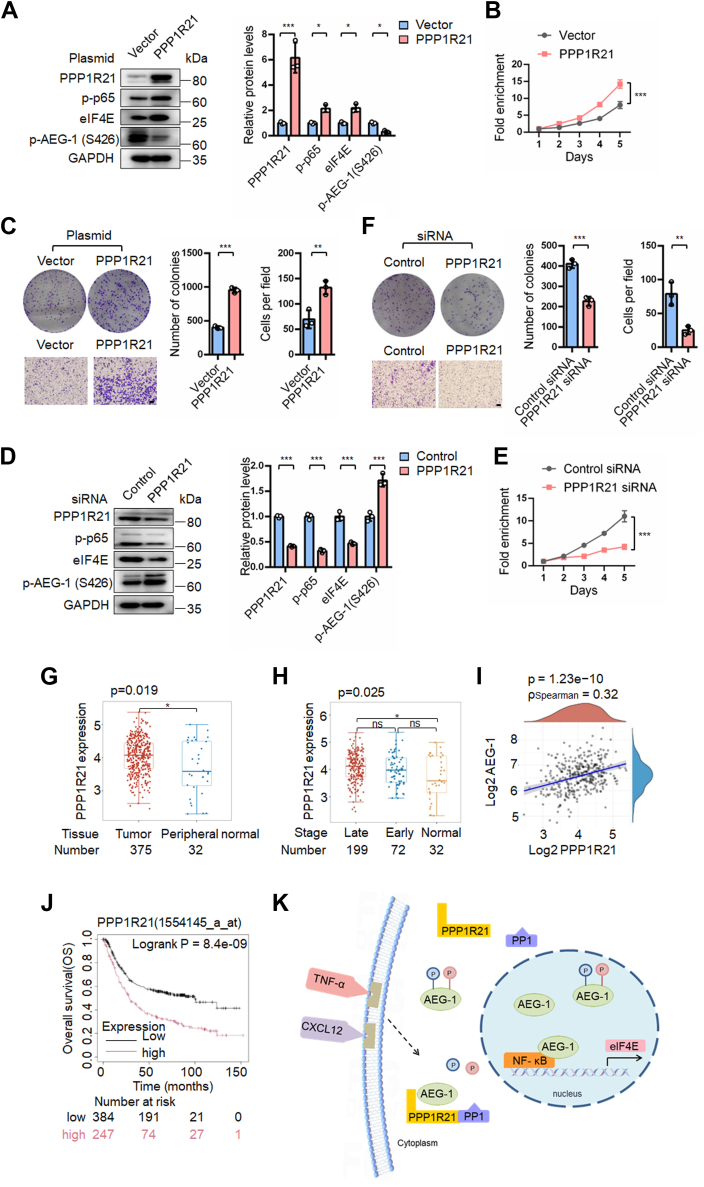
**PPP1R21 induced dephosphorylation of AEG-1 S426 to enhance its pro-oncogenic function in gastric cancer**. *A*–*C*, BGC823 cells were transfected with PPP1R21 overexpression plasmids or control vector. *D*–*F*, BGC823 cells were transfected with PPP1R21 siRNAs or control siRNAs. Cells were then subjected to Western blotting, and SRB assay, colony formation assay, and transwell migration assay. Western blot bands were quantified by densitometry, and values were normalized to GAPDH. Data are presented as mean ± SD from three independent experiments. Points, means of four replicate determinations (SRB); columns, means of three replicate determinations (colony formation); columns, means of three microscopic fields (transwell); the bars represent SD. ∗∗*p* < 0.01 and ∗∗∗*p* < 0.001. The scale bar represents 200 μm. *G* and *H*, bioinformatics analysis of PPP1R21 expression in gastric cancer tissues based on TCGA datasets using the Home for Researchers platform (https://www.home-for-researchers.com). *I*, the correlation between PPP1R21 and AEG-1 expression in gastric cancer, based on TCGA datasets, analyzed using the Home for Researchers platform. *J*, correlation between PPP1R21 expression and patient survival, analyzed using the Kaplan–Meier Plotter tool (http://kmplot.com/analysis). *K*, schematic diagram illustrating the mechanism by which AEG-1 phosphorylation regulates eIF4E expression. CXCL12 or TNF-α promotes the binding of PPP1R21 to AEG-1, leading to its dephosphorylation at S308 and S426 by PP1. In the nucleus, dephosphorylated AEG-1 interacts with NF-κB to enhance its transcriptional activity, thereby activating the eIF4E promoter and upregulating eIF4E expression. AEG-1, astrocyte elevated gene-1; CXCL12, CXC chemokine ligand 12; eIF4E, eukaryotic translation initiation factor 4E; PP1, protein phosphatase 1; PPP1R21, protein phosphatase 1 regulatory subunit 21; S308, serine 308; S426, serine 426; SRB, sulforhodamine B; TCGA, The Cancer Genome Atlas; TNF-α, tumor necrosis factor alpha.

We next examined the effects of PPP1R21 on AEG-1 phosphorylation. Western blot analysis showed that overexpression of PPP1R21 reduced p-AEG-1 (S426) levels, whereas PPP1R21 knockdown increased p-AEG-1 (S426) levels (Fig. 7, *A* and *D*). Overexpression of PPP1R21 significantly upregulated eIF4E and p-p65 NF-κB protein levels and promoted cell growth and migration (Fig. 7, *A*–*C*). In contrast, knockdown of PPP1R21 using siRNAs suppressed these effects (Fig. 7, *D*–*F*). These results suggest that PPP1R21 facilitates AEG-1 dephosphorylation and activation, thereby enhancing the oncogenic functions of AEG-1.

Currently, the expression profile of PPP1R21 in human cancers remains largely unexplored. To evaluate its relevance in gastric cancer, we analyzed the expression of PPP1R21 using the Home for Researcher platform based on The Cancer Genome Atlas datasets. PPP1R21 expression was significantly higher in gastric cancer tissues than in normal tissues and tended to increase disease progression, as we observed higher expression levels in late-stage cancers (stages III and IV) than in early-stage cancers (stages Ib and IIa), although this difference did not reach statistical significance (Fig. 7, *G* and *H*). Moreover, PPP1R21 expression levels positively correlated with AEG-1 expression (Fig. 7*I*). Importantly, high PPP1R21 expression was associated with poor overall survival in gastric cancer patients (Fig. 7*J*).

Collectively, these findings suggest that PPP1R21 is upregulated in gastric cancer, promotes AEG-1 dephosphorylation, and contributes to cancer progression and poor patient prognosis.

## Discussion

AEG-1 plays an important role in the progression of various cancers; however, its post-translational modifications, such as phosphorylation, remain largely unexplored. To date, only one MS study by Krishnan *et al.* (33) has revealed direct p hosphorylation of AEG-1 at S298 by inhibitory kappa B kinase beta in response to TNF-α stimulation. Follow-up work by the same group demonstrated that phosphorylation at S298 is oncogenic, promoting breast cancer cell growth through studies using AEG-1 S298 mutant plasmids (S298A and S298D) (26). In the present study, we identified two novel phosphorylation sites of AEG-1, S426 and S308, in gastric cancer. In contrast to Krishnan’s findings, we revealed that the phosphorylation at S308 and S426 renders AEG-1 inactive and suppresses the growth and metastasis of gastric cancer both *in vitro* and *in vivo*. Conversely, dephosphorylated AEG-1 represents its active form and can be induced by CXCL12 or TNF-α. This discrepancy is not surprising, as phosphorylation at different sites may result in either activation or inactivation of a protein, depending on how it affects protein conformation, downstream interactions, stability, or the post-translational modifications of other amino acid residues. Recently, palmitoylation of AEG-1 at cysteine 75 was reported to induce protein degradation and disrupt its interaction with SND1 (34). Our study only identifies two phosphorylation sites; therefore, further studies on the phosphorylation of other residues are expected to provide further insight into its regulatory mechanisms in cancer.

We previously reported that AEG-1 promotes the growth and metastasis of gastric cancer by upregulation of eIF4E expression; however, the underlying mechanism remained unclear. In the present study, we demonstrated that dephosphorylation of AEG-1 (active state) activates the eIF4E promoter and upregulates its transcription. Several potential transcription factors that may bind to the eIF4E promoter were identified by bioinformatics analysis, among which NF-κB, c-Fos, and C/EBP attracted our attention. AEG-1 is well known to interact with p65 NF-κB, promote its nuclear translocation, and activate the promoters of target genes, such as *c-Fos* and *IL-8*. In this study, we found that dephosphorylation of AEG-1 at S308, but not at S426, activates p65 NF-κB and increases *c-Fos* expression. Our findings support a model in which S308 dephosphorylation facilitates the transcriptional activation of eIF4E driven by S426 dephosphorylation. To understand the interplay between phosphorylation of S308 and S426, we examined the p-AEG-1 S426 levels in cells with AEG-1 S308A or S308D overexpression by Western blotting. To our surprise, no obvious changes were observed (Fig. S5*C*). Considering that this result might be caused by insufficient endogenous PPP1R21 levels to dephosphorylate the overexpressed AEG-1 proteins (WT, S426A, or S426D), we repeated this experiment with cotransfection of PPP1R21 plasmids. Western blotting showed that AEG-1 S308D overexpression increased p-AEG-1 S426 levels, whereas S308A decreased them (Fig. S5*D*). These findings suggest that S308 phosphorylation may enhance S426 phosphorylation. However, it remains unclear whether, and how, S308 dephosphorylation may influence the binding of AEG-1 to other regulatory proteins, such as transcription factors (*e.g.*, C/EBP) or histone acetyltransferases (*e.g.*, p300), which are known to regulate eIF4E transcription. AEG-1 has previously been shown to form a complex with p300 and c-Jun, facilitating the acetylation and activation of c-Jun and promoting downstream gene transcription in glioma cells (35). In addition, our previous work in lung cancer revealed that the eIF4E promoter was activated epigenetically by histone acetylation, rather than direct transcription factor binding (36). Given that AEG-1 functions as a scaffold protein through interactions with various partners, our findings suggest that AEG-1 phosphorylation may regulate its protein–protein interactions, most likely by altering its three-dimensional conformation.

A novel finding of this study is that AEG-1 is dephosphorylated by PP1 through binding to the C-terminal region of PPP1R21. It is well established that some serine/threonine phosphatases, such as members of the PP1 and PP2 families, function as multimeric complexes composed of a catalytic subunit and one or more regulatory subunits, whereas other phosphatases, such as members of the PP3 (calcineurin) family, contain both catalytic and regulatory domains within the same polypeptide (19, 20). Based on our co-IP results and subsequent MS analysis in a separate project in our laboratory, we selected PPP1R21 for further study because structural predictions indicated it to be a potential regulatory subunit of PP1. A previous study identified 78 novel regulatory subunits of PP1 through *in silico* screening based on a stringent definition of the RVxF motif, among which PPP1R21 was experimentally validated (33). However, studies on the biological function of PPP1R21 remain very limited. To date, aberrant PPP1R21 has been implicated in syndromic neurodevelopmental disorders, as observed in a cohort of 13 affected individuals (37, 38). Although PPP1R21 has been reported to participate in endosomal maturation, the precise molecular mechanisms are not elucidated (39).

Currently, the expression and function of PPP1R21 in cancer are unknown. In this study, we found that elevated expression of PPP1R21 correlates with gastric cancer progression and poor patient survival. Functionally, PPP1R21 promotes the growth and migration of gastric cancer cells. Mechanistically, CXCL12 induces the binding of PPP1R21 with AEG-1, thereby promoting PPP1R21-mediated PP1 dephosphorylation of AEG-1 at S426. However, the mechanism by which CXCL12 triggers this interaction remains unclear. Given that PPP1R21 contains potential phosphorylation sites, it is possible that CXCL12 activates a downstream kinase that phosphorylates PPP1R21, altering its substrate-binding properties. Although drug development targeting phosphatase holoenzymes was abandoned a decade ago because of specificity challenges, new allosteric molecules that disrupt the interaction between catalytic and regulatory subunits have shed light on this field (21). For example, the small molecule Raphin 1 selectively targets PPP1R15B, whereas Guanabenz and Sephin 1 target PPP1R15A, to inhibit PP1 function in specific pathological contexts (17). These findings suggest that PPP1R21 may play an important role in the development of gastric cancer and neurodevelopmental disorders. Therefore, PPP1R21 is a potential therapeutic target and worthy of future investigation.

We and others have reported that elevated expression of AEG-1 is correlated with cancer progression and poor prognosis, including in gastric cancer (5). However, the phosphorylation status of AEG-1 in human tissue samples is quite unknown. In the present study, we developed a specific antibody to detect phosphorylation of AEG-1 at S426. This antibody successfully identified phosphorylated AEG-1 in both gastric cancer cell lines and human tissue samples. Further analysis revealed that decreased p-AEG-1 S426 levels were associated with poor pathological characteristics of gastric cancer, such as lymph node metastasis and advanced pTNM stage. These findings confirm the existence of phosphorylated AEG-1 in gastric cancer and suggest that decreased p-AEG-1 S426 levels may serve as a potential novel adjuvant diagnostic marker, in addition to total AEG-1 expression.

In summary, we found that AEG-1 undergoes dephosphorylation at S426 and S308 in gastric cancer in response to upstream stimuli such as CXCL12 and TNF-α. This process is mediated by PPP1R21 through its interaction with PP1. Dephosphorylation at either or both sites renders AEG-1 functionally active and promotes gastric cancer progression by upregulating eIF4E expression and NF-κB signaling. p-AEG-1 S426 levels decrease during disease progression and are associated with poor prognosis. Our findings identify phosphorylation of AEG-1 S426 as a potential new diagnostic marker and a potential therapeutic target in gastric cancer.

## Experimental procedures

### Reagents and antibodies

Recombinant Human TNF-alpha Protein (RP00001) and CXCL12 protein (RP00201) were obtained from ABclonal Biotechnology. Lipofectamine 2000 Transfection Reagent (11668019) was purchased from Life Technologies Co, Invitrogen. The antibodies anti-Phospho-NF-κB p65 (Ser536) (93H1), anti-NF-κB p65 (#8242), and anti-eIF4E (#9742) were obtained from Cell Signaling Technology; anti-AEG-1 (13860-1-AP), anti-Myc-tag (60003-2-lg), and mouse IgG (B900620) were obtained from Proteintech Group; anti-GAPDH (AP0063), anti-HA-tag (4G3) (AP0005M), and anti-FLAG-tag (1A8) (AP0007MP) were obtained from Bioworld Technology; anti-PPP1CA (A24288) was obtained from ABclonal Biotechnology. Rabbit IgG horseradish peroxidase–conjugated antibody (HAF008) and mouse IgG horseradish peroxidase–conjugated antibody (HAF018) were obtained from R&D Systems. Customized antibodies, anti-p-AEG-1 (S426) and anti-PPP1R21, were developed by Abmart Shanghai Co, Ltd and Shanghai HuiOu Biotech Co, Ltd, respectively.

### Cell lines

The human gastric cancer cell lines (BGC823, MGC803, MKN45, and SGC7901), the human normal gastric mucosal cell line GES-1, and the human embryonic kidney cell line HEK293T were obtained from the American Type Culture Collection. Cells were cultured in Dulbecco's modified Eagle's medium supplemented with 10% fetal bovine serum and maintained in an incubator at 37 °C with 5% CO_2_. All cell lines were authenticated by profiling 20 short tandem repeats and tested periodically to exclude mycoplasma contamination using Hoechst staining.

### Human tissue samples and IHC staining

All studies involving human tissue were approved by the Ethics Committee of Nanjing Medical University and conducted in accordance with the Declaration of Helsinki. Informed consent was obtained from all participants. All experiments were performed following the approved guidelines of Nanjing Medical University.

Eighty-five gastric cancer tissue samples were collected from patients who underwent surgical resection at Nanjing First Hospital, Nanjing Medical University, between 2015 and 2020. None of the patients received treatment before the resection. Pathological diagnoses were made according to the World Health Organization Classification of Digestive System Tumor (fifth edition). Paraffin-embedded tissue sections were subjected to IHC staining using antibodies specific for phosphorylated AEG-1 at S426. Each slide was evaluated independently by at least two experienced pathologists. IHC staining and scoring were performed as previously described (40).

### Gene overexpression plasmid construction

Previously constructed HA-tagged AEG-1 WT plasmids in the pcDNA3.1 vector (HA-AEG-1 WT) were used as templates. Site-directed mutagenesis was performed using the Mut Express Fast Mutagenesis kit (catalog no.: C214-01) from Vazyme Biotech Co, Ltd to generate AEG-1 point mutants, including S426A, S426D, S308A, and S308D. The AEG-1 double mutants (S308/426A and S308/426D) were generated using S426A or S426D plasmids as templates by introducing a second point mutation. FLAG-tagged PPP1R21 overexpression plasmids (FLAG-PPP1R21) were constructed by inserting full-length PPP1R21 (NM_001135629) into the p3×FLAG CMV14 vector. Truncated PPP1R21 plasmids were generated by amplifying the N-terminal or C-terminal regions of PPP1R21 *via* PCR, using the full-length FLAG-PPP1R21 construct as a template. Myc-tagged PP1cs overexpression plasmids (Myc-PP1cs) were constructed by inserting full-length PP1cs (NM_002708.4) into the pCMV5-Myc vector.

### Gene knockdown and overexpression

siRNAs targeting AEG-1 and PPP1R21 were pools of two or three independent sequences targeting each gene. These siRNAs and control siRNAs (nontarget siRNAs) were synthesized by GenePharma Co, Ltd. The siRNAs and plasmids were delivered into cells using Lipofectamine 2000 following the manufacturer’s instructions. The siRNA sequences were as follows:

AEG-1 siRNA sequence 1: 5′-CAG AAGAAGAAGAACCGG A-3′;

AEG-1 siRNA sequence 2: 5′-GCAGCAAGGCAGUCUUUAAGU-3′;

PPP1R21 siRNA sequence 1: 5′-GCA GAACGACAAGGCUAAA-3′;

PPP1R21 siRNA sequence 2: 5′-GCAACACAGAAGCUGAUAA-3′;

PPP1R21 siRNA sequence 3: 5′-GCAGCGAGUGGAUUCAUUA-3′.

BGC823 cells stably overexpressing WT or S308/426D double mutant AEG-1 were generated by transducing lentiviruses carrying the corresponding AEG-1 coding sequences packaged by GeneChem Co, Ltd.

### Dual-luciferase reporter assay

The eIF4E promoter luciferase plasmid was constructed by inserting the −1507 to +72 bp region of the human eIF4E promoter (pGL3-eIF4E-promoter) into the pGL3-Basic vector, which was a kind gift from Dr Shi-Yong Sun at Emory University (41). NF-κB transcriptional activity was measured using the pNF-κB-luc plasmid (catalog no.: D2206) purchased from Beyotime Biotechnology Co, Ltd. This plasmid contains four tandem NF-κB response elements inserted into the pGL6 vector. The pRL-TK plasmid encoding Renilla luciferase was cotransfected as an internal control. Luciferase activity was measured using the Dual-Luciferase Reporter Gene Assay Kit from Yeason Biotechnology Co, Ltd. Firefly luciferase signals were normalized to Renilla luciferase activity.

### Xenograft and peritoneal metastasis mouse models

Animal experiments were approved by the Institutional Animal Care and Use Committee of the Nanjing Medical University and followed the institutional guidelines. Female BALB/c nude mice (4–6 weeks old) were purchased from GemPharmatech Co, Ltd and housed under standard conditions at the Animal Core Facility of Nanjing Medical University. For the xenograft model, mice were inoculated subcutaneously with BGC823 cells stably expressing WT or mutant AEG-1 (5 × 10^6^ cells/mouse). Tumor volume was calculated using the formula: π (length × width^2^)/6. Tumors were harvested and stored at −80 °C for qRT–PCR and Western blot analysis. For the peritoneal metastasis model, BGC823 cells (1 × 10^5^ cells/mouse) were injected intraperitoneally. After 4 weeks, metastatic nodules on the surfaces of the liver and intestinal tract were examined and counted.

### IP, MS analysis, and Western blotting

Whole-cell protein lysates were prepared using 0.5% CHAPS lysis buffer for IP or 1% Triton X-100 lysis buffer for Western blot. Pulled-down proteins were separated by SDS-PAGE and visualized *via* silver nitrate staining. The full lane was excised and subjected to MS analysis at the Nanjing Medical University, as previously described (40). The mass spectrometry raw data have been deposited in the PRIDE database and will be provided upon request.

Western blot quantification was performed as follows. For each target protein band, the intensity was calculated as the ratio of the area value of the target band to that of the housekeeping protein band. Fold changes between treatment and control groups were calculated using the formula: fold change = (value ratio of treatment)/(value ratio of control), and the results were presented as column charts. Data are shown as mean ± SD from three independent experiments.

### RNA isolation and qRT–PCR

Total RNA isolation, qRT–PCR, and data analysis were performed as described previously (40). All primers were synthesized by Tsingke Biotechnology with sequences as below:

Human *IL-8*: Forward: 5′-TGCCAAGGAGTGCTAAAG-3′,

Reverse: 5′-TCTCAGCCCTCTTCAAAA-3′;

Human *FOS*: Forward: 5′-TAGCAAAACGCATGGAGTGT-3′,

Reverse: 5′-GCCTGGCTCAACATGCTACT-3′;

Human *eIF4E*: Forward: 5′-CCTACAGAACAGATGGGCACTC-3′,

Reverse: 5′-GCCCAAAAGTCTTCAACAGTATCA-3′;

Human *GAPDH*: Forward: 5′-ATGGGGAAGGTGAAGGTCG-3′,

Reverse: 5′-GGGGTCATTGATGGCAACAACAATA-3′.

### Nuclear and cytoplasmic protein separation

Nuclear and cytoplasmic proteins were separated using a Nuclear and Cytoplasmic Protein Extraction Kit (P0028; Beyotime Biotechnology) following the manufacturer's instructions.

### SRB assay, transwell migration assay, and invasion assay

SRB assay, transwell migration assay, and Matrigel-based invasion assay were conducted as we previously reported (40).

### Statistical analysis

Statistical analyses were conducted using GraphPad Prism 6.0 (GraphPad Software, Inc). Normality and homogeneity of variance were assessed for all datasets. For two-group comparisons, either a two-tailed unpaired Student’s *t* test or the Mann–Whitney *U* test was used, depending on whether the data met the assumptions of normality and equal variance. For multiple group comparisons, one-way ANOVA followed by Tukey’s *post hoc* test or the Kruskal–Wallis test was applied accordingly. Data were presented as mean ± SD, and *p <* 0.05 was considered statistically significant. Detailed statistical methods and corresponding *p* values are provided in File S3.

## Data availability

The data generated in this study are available within the article and its supporting information files.

## Supporting information

This article contains supporting information (supporting figures and supporting files).

## Conflict of interest

The authors declare that they have no conflicts of interest with the contents of this article.
