# Supplementary material for: Dephosphorylation of astrocyte elevated gene-1 protein upregulates eIF4E expression to promote gastric cancer progression
Source: J Biol Chem. 2025 Sep 4;301(10):110687. doi: 10.1016/j.jbc.2025.110687 (PMC12519287; doi:10.1016/j.jbc.2025.110687)
Supplement: Supplementary figures [file mmc1.pdf]

Dephosphorylation of astrocyte elevated gene-1 protein upregulates eIF4E expression to promote gastric cancer progression

Li Zhao, Xixi Qian, Yaoyao Fan, Huiling Li, Zuhao, Zhang, Chen Chen, Lulu Yang, Shaorong Yu*, Xuerong Wang*, Wenbin Huang*

Supplementary Figures

Supplementary Figure S1

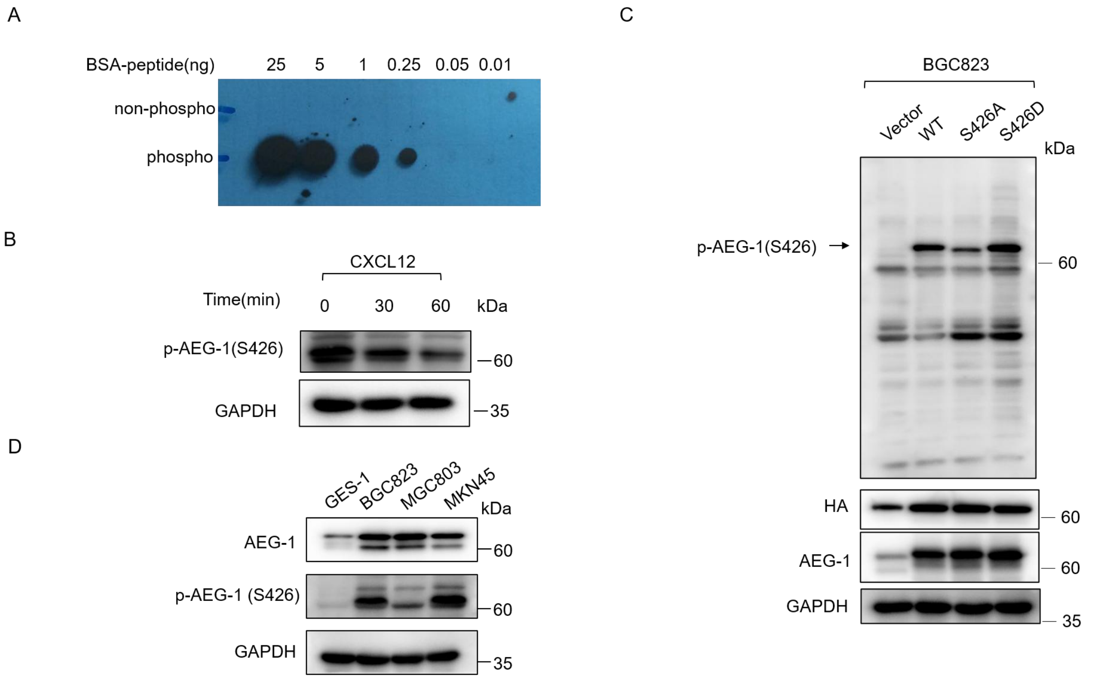

Supplementary Figure S1 Phosphorylation of AEG-1 S426 was detectable in gastric cancer cell lines and human tissues. A, dot blot validation of the anti–p-AEG-1 (S426) antibody. Phosphorylated and non-phosphorylated peptides corresponding to the S426 site were spotted onto nitrocellulose membranes. The antibody was applied at serial dilutions as indicated. HRP-conjugated secondary antibodies and ECL substrate were used for detection. B, SGC7901 cells were treated with 100 ng/mL CXCL12 for 30 or 60 min and subjected to Western blotting. C, BGC823 cells were transfected with AEG-1 WT, S426A, or S426D plasmids for 48 hours and subjected to Western blotting. D, gastric cancer cell lines and normal gastric mucosal cells were cultured for 48 hours and subjected to Western blotting. The specific p-AEG-1 (S426) band is indicated by a black arrow.

Supplementary Figure S2

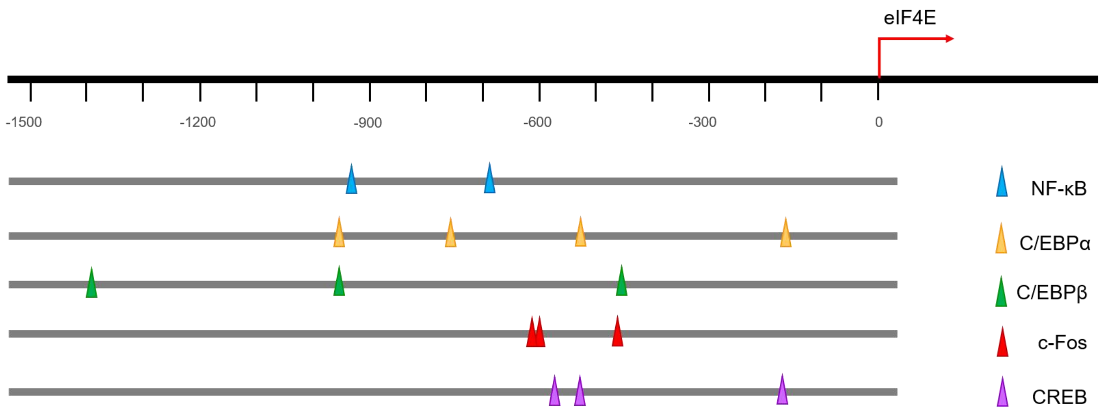

Transcription factors	NF-κB	C/EBP α	C/EBP β	c-Fos	CREB
position	-689 to -680bp	-175 to -161bp	-460 to -452bp	-459 to -452bp	-179 to -172bp
	-945 to -936bp	-526 to -516bp	-968 to -959bp	-605 to -598bp	-525 to -518bp
		-767 to -757bp	-1399 to -1390bp	-619 to -610bp	-583 to -576bp
		-968 to -959bp			

Supplementary Figure S2 A schematic diagram of transcription factors predicted to bind the eIF4E promoter. Bioinformatic analysis of the *eIF4E* promoter region was performed using the JASPAR platform (https://jaspar.elixir.no/) and the Human Transcription Factor Database (human TFDB; http://bioinfo.life.hust.edu.cn/HumanTFDB/#!/) to identify potential transcription factor binding sites.

Supplementary Figure S3

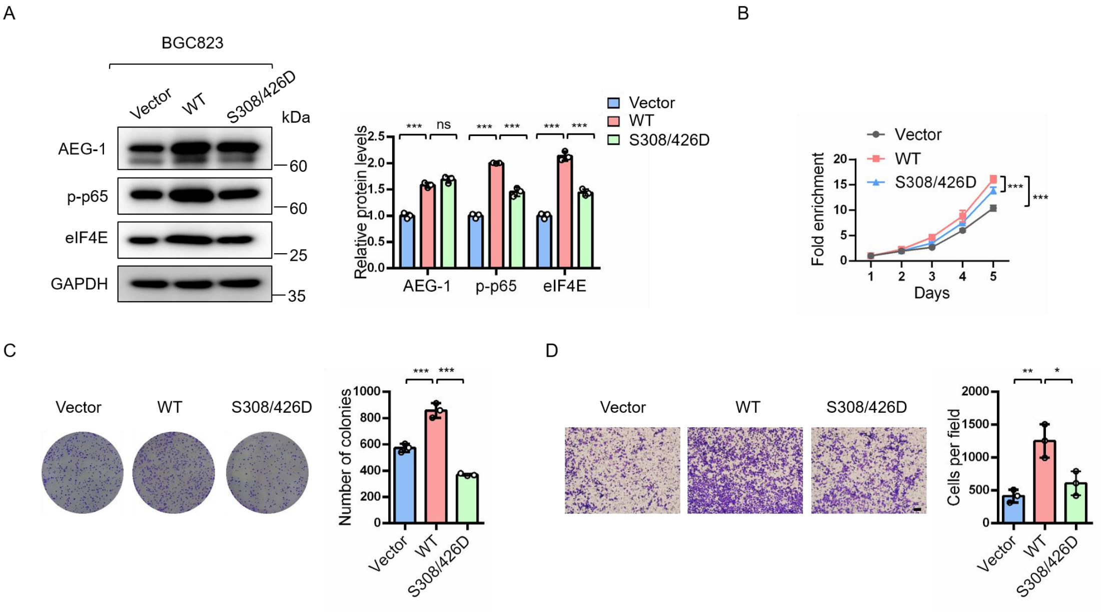

Supplementary Figure S3 Stable overexpression of AEG-1 S308/426D downregulated eIF4E expression and NF-κB signaling, and suppressed gastric cancer cell growth and migration compared to AEG-1 WT. BGC823 cells stably overexpressing AEG-1 WT, S308/426D mutant, or vector were subjected to Western blot analysis (A), SRB assay (B), colony formation assay (C), and transwell migration assay (D). Western blot bands were quantified by densitometry, and values were normalized to GAPDH. Data are presented as mean ± SD from three independent experiments. Points, means of four replicate determinations (SRB); columns, means of three replicate determinations (colony formation); columns, means of three microscopic fields (transwell); bars, SD. *, $P < 0.05$; **, $P < 0.01$; ***, $P < 0.001$. Scale bar: 200 μm.

Supplementary Figure S4

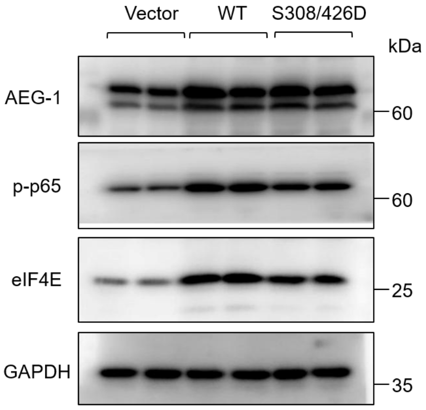

Supplementary Figure S4 Expression of eIF4E and p-p65 NF-κB in the xenografts of mice with dual phosphorylation of AEG-1 at S308/426. Protein lysates of xenografts were prepared and subjected to Western blotting. Representative Western blot image from two mice per group were shown (Western blot image from the other three mice in each group were shown in Fig. 5D).

Supplementary Figure S5

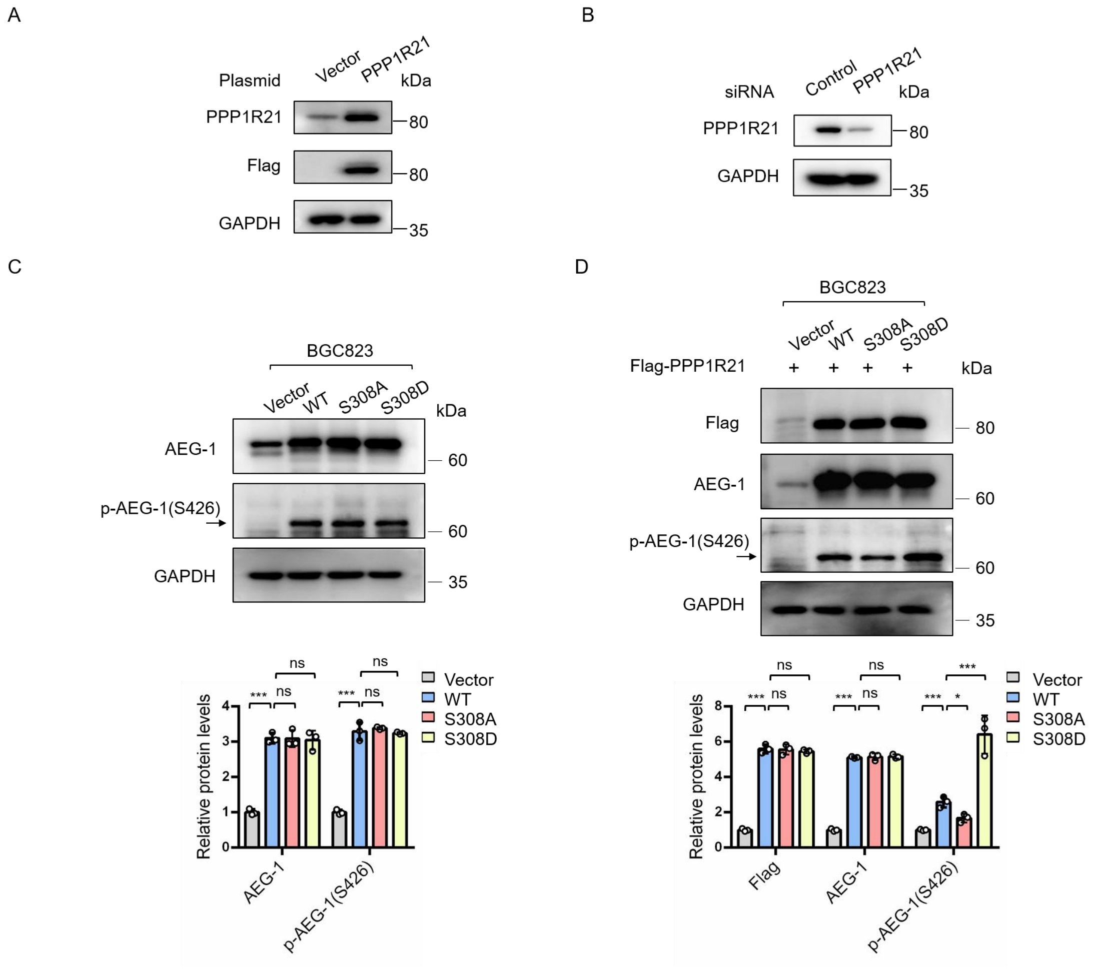

Supplementary Figure S5 Validation of the anti-PPP1R21 antibody and evidence that AEG-1 S308 phosphorylation promotes AEG-1 S426 phosphorylation. A, BGC823 cells were transfected with Flag-PPP1R21 overexpression plasmids or vector control for 48 hours. B, BGC823 cells were transfected with a pool of siRNAs targeting PPP1R21 or non-targeting control siRNAs for 48 hours. Whole-cell lysates were prepared and subjected to Western blotting. C, BGC823 cells were transfected with AEG-1 WT, S308A, or S308D plasmids for 48 hours and subjected to Western blotting. D, BGC823 cells were co-transfected with PPP1R21 plasmids and either AEG-1 WT or mutant plasmids as indicated, followed by Western blotting. Western blot bands were quantified by densitometry, and values were normalized to GAPDH. Data are presented as mean ± SD from three independent experiments. *, $P < 0.05$; **, $P < 0.01$; ***, $P < 0.001$.
