# Supplementary material for: Dephosphorylation of astrocyte elevated gene-1 protein upregulates eIF4E expression to promote gastric cancer progression
Source: J Biol Chem. 2025 Sep 4;301(10):110687. doi: 10.1016/j.jbc.2025.110687 (PMC12519287; doi:10.1016/j.jbc.2025.110687)
Supplement: Supplementary file S3 [file mmc4.doc]

Statistical Analysis

| **Comparison Group** | **Mean ± SD** | **Statistical Test** | **p-value** |
| --- | --- | --- | --- |
| Late vs Early | 3.72 **±** 4.68 ; 6.6 ± 4.43 | Mann-Whitney test | P=0.0005 |

**Figure1G**

**Figure1H**

| **Comparison Group** | **Mean ± SD** | **Statistical Test** | **p-value** |
| --- | --- | --- | --- |
| Metastasis vs no Metastasis | 3.78±4.402 ; 5.954±4.9 | Mann-Whitney test | P=0.0456 |

**Figure2 A**

| **Comparsion Group** | | | **Mean ± SD** | **Statistical Test** | **p-value** |
| --- | --- | --- | --- | --- | --- |
| BGC823 | AEG-1 | WT vs S426A | 1 ± 0.2197 ; 1.017 ± 0.1587 | one-way ANOVA | >0.9999 |
|  |  | WT vs S426D | 1 ± 0.2197 ; 1.003 ± 0.1877 | one-way ANOVA | >0.9999 |
|  | eIF4E | WT vs S426A | 1 ± 0.1281 ; 2.365 ± 0.2947 | one-way ANOVA | <0.0001 |
|  |  | WT vs S426D | 1 ± 0.1281; 0.3991± 0.07838 | one-way ANOVA | 0.0011 |
|  | p-p65 | WT vs S426A | 1 ± 0.1885; 0.9489 ± 0.1009 | one-way ANOVA | >0.9999 |
|  |  | WT vs S426D | 1 ± 0.1885; 1.002 ± 0.1067 | one-way ANOVA | >0.9999 |
| MGC803 | AEG-1 | WT vs S426A | 1 ± 0.2094; 0.9705 ± 0.1506 | one-way ANOVA | =0.9499 |
|  |  | WT vs S426D | 1 ± 0.2094; 0.9778 ± 0.07925 | one-way ANOVA | =0.9711 |
|  | eIF4E | WT vs S426A | 1 ± 0.1548; 1.799 ± 0.1114 | one-way ANOVA | <0.0001 |
|  |  | WT vs S426D | 1 ± 0.1548; 0.4617 ± 0.06785 | one-way ANOVA | =0.0003 |
|  | p-p65 | WT vs S426A | 1 ± 0.1203; 1.0461 ± 0.166 | one-way ANOVA | =0.8836 |
|  |  | WT vs S426D | 1 ± 0.1203; 1.021 ± 0.1035 | one-way ANOVA | =0.9742 |
| MKN45 | AEG-1 | WT vs S426A | 1 ± 0.1149; 1.031 ± 0.2903 | one-way ANOVA | =0.9695 |
|  |  | WT vs S426D | 1 ± 0.1149; 1.028 ± 0.2025 | one-way ANOVA | =0.9743 |
|  | eIF4E | WT vs S426A | 1 ± 0.1767; 1.605 ± 0.2204 | one-way ANOVA | =0.0014 |
|  |  | WT vs S426D | 1±0.1767; 0.254 ± 0.1188 | one-way ANOVA | =0.0002 |
|  | p-p65 | WT vs S426A | 1±0.1339; 1.134 ± 0.03482 | one-way ANOVA | =0.5803 |
|  |  | WT vs S426D | 1±0.1339; 0.9803 ± 0.2219 | one-way ANOVA | =0.9873 |

**Figure2 B**

| **Comparsion Group** | | **Mean ± SD** | **Statistical Test** | **p-value** |
| --- | --- | --- | --- | --- |
| BGC823 | WT vs Vector | 2.61±0.4702; 1±0 | one-way ANOVA | <0.0001 |
|  | WT vs S426A | 2.61±0.4702; 3.84±0.52 | one-way ANOVA | <0.0001 |
|  | WT vs S426D | 2.61±0.4702; 1.23±0.1805 | one-way ANOVA | <0.0001 |
| MGC803 | WT vs Vector | 2.16±0.22; 1±0 | one-way ANOVA | <0.0001 |
|  | WT vs S426A | 2.16±0.22; 3.12±0.2163 | one-way ANOVA | =0.0007 |
|  | WT vs S426D | 2.16±0.22; 0.78±0.2797 | one-way ANOVA | <0.0001 |
| MKN45 | WT vs Vector | 2.625±0.316; 1±0 | one-way ANOVA | <0.0001 |
|  | WT vs S426A | 2.625±0.316; 3.594±0.2756 | one-way ANOVA | =0.0006 |
|  | WT vs S426D | 2.625±0.316; 0.5824±0.1592 | one-way ANOVA | <0.0001 |

**Figure2 C**

| **Comparsion Group** | | **Mean ± SD** | **Statistical Test** | **p-value** |
| --- | --- | --- | --- | --- |
| BGC823 | WT vs Vector | 4.727±0.2786 ; 1±0 | one-way ANOVA | <0.0001 |
|  | WT vs S426A | 4.727±0.2786 ; 7.05±0.24 | one-way ANOVA | <0.0001 |
|  | WT vs S426D | 4.727±0.2786 ; 2.868±0.246 | one-way ANOVA | <0.0001 |
| MGC803 | WT vs Vector | 4.528±0.2993 ; 1±0 | one-way ANOVA | <0.0001 |
|  | WT vs S426A | 4.528±0.2993 ; 7.95±0.5389 | one-way ANOVA | <0.0001 |
|  | WT vs S426D | 4.528±0.2993 ; 2.666±0.2359 | one-way ANOVA | <0.0001 |
| MKN45 | WT vs Vector | 4.123±0.4211 ; 1±0 | one-way ANOVA | <0.0001 |
|  | WT vs S426A | 4.123±0.4211 ; 8.521±0.5134 | one-way ANOVA | <0.0001 |
|  | WT vs S426D | 4.123±0.4211 ; 2.568±0.3434 | one-way ANOVA | <0.0001 |

**Figure2 D**

| **Comparsion Group** | | **Mean ± SD** | **Statistical Test** | **p-value** |
| --- | --- | --- | --- | --- |
| BGC823 | WT vs Vector | 6.321±0.6019 ; 1±0 | one-way ANOVA | <0.0001 |
|  | WT vs S426A | 6.321±0.6019 ; 6.541±0.1471 | one-way ANOVA | 0.4917 |
|  | WT vs S426D | 6.321±0.6019 ; 6.213±0.1732 | one-way ANOVA | 0.8820 |
| MGC803 | WT vs Vector | 5.46±0.1465 ; 1±0 | one-way ANOVA | <0.0001 |
|  | WT vs S426A | 5.46±0.1465 ; 5.231±0.1632 | one-way ANOVA | 0.4607 |
|  | WT vs S426D | 5.46±0.1465 ; 5.114±0.3001 | one-way ANOVA | 0.1652 |
| MKN45 | WT vs Vector | 4.713±0.09904 ; 1±0 | one-way ANOVA | <0.0001 |
|  | WT vs S426A | 4.713±0.09904 ; 4.617±0.1497 | one-way ANOVA | 0.9132 |
|  | WT vs S426D | 4.713±0.09904 ; 4.863±0.0982 | one-way ANOVA | 0.7477 |

**Figure2 E**

| **Comparsion Group** | | **Mean ± SD(5 Days)** | **Statistical Test** | **p-value** |
| --- | --- | --- | --- | --- |
| BGC823 | WT vs S426A | 9.378±0.2312 ; 10.96±0.1712 | one-way ANOVA | <0.0001 |
|  | WT vs S426D | 9.378±0.2312 ; 7.918±0.214 | one-way ANOVA | <0.0001 |
| MGC803 | WT vs S426A | 12.299±1.121 ; 15.411±0.6159 | one-way ANOVA | <0.0001 |
|  | WT vs S426D | 12.299±1.121 ; 10.52±0.516 | one-way ANOVA | <0.0001 |
| MKN45 | WT vs S426A | 13.06±0.1568 ; 16.35±0.4018 | one-way ANOVA | <0.0001 |
|  | WT vs S426D | 13.06±0.1568 ; 10.79±0.5152 | one-way ANOVA | <0.0001 |

**Figure2 F**

| **Comparsion Group** | | **Mean ± SD** | **Statistical Test** | **p-value** |
| --- | --- | --- | --- | --- |
| BGC823 | WT vs S426A | 557±31.22 ; 706±63.65 | Kruskal-Wallis test | 0.0034 |
|  | WT vs S426D | 557±31.22 ; 272±66.64 | Kruskal-Wallis test | <0.0001 |
| MKN45 | WT vs S426A | 540±29.46 ; 765.7±38.94 | Kruskal-Wallis test | 0.0001 |
|  | WT vs S426D | 540±29.46 ; 382±27.22 | Kruskal-Wallis test | 0.0022 |

**Figure2 G**

| **Comparsion Group** | | **Mean ± SD** | **Statistical Test** | **p-value** |
| --- | --- | --- | --- | --- |
| BGC823 | WT vs S426A | 623±28.48 ; 959±139 | Kruskal-Wallis test | 0.0014 |
|  | WT vs S426D | 623±28.48 ; 221±23.25 | Kruskal-Wallis test | 0.0003 |
| MKN45 | WT vs S426A | 574±42.51 ; 856±159.6 | Kruskal-Wallis test | 0.0051 |
|  | WT vs S426D | 574±42.51 ; 304±51.1 | Kruskal-Wallis test | 0.0068 |

**Figure3 A**

| **Comparsion Group** | | | **Mean ± SD** | **Statistical Test** | **p-value** |
| --- | --- | --- | --- | --- | --- |
| BGC823 | AEG-1 | WT vs S308A | 1±0.1808 ; 1.012±0.08305 | Kruskal-Wallis test | 0.9920 |
|  |  | WT vs S308D | 1±0.1808 ; 0.9837±0.2056 | Kruskal-Wallis test | 0.9861 |
|  | p-p65 | WT vs S308A | 1±0.1993 ;1.643±0.1877 | Kruskal-Wallis test | <0.0001 |
|  |  | WT vs S308D | 1±0.1993 ; 0.6167±0.1187 | Kruskal-Wallis test | 0.0064 |
|  | p65 | WT vs S308A | 1±0.06644 ; 1.043±0.05616 | Kruskal-Wallis test | 0.9089 |
|  |  | WT vs S308D | 1±0.06644 ; 1.071±0.06588 | Kruskal-Wallis test | 0.7715 |
|  | eIF4E | WT vs S308A | 1±0.01295 ; 1.663±0.2434 | Kruskal-Wallis test | <0.0001 |
|  |  | WT vs S308D | 1±0.01295 ; 0.5969±0.08344 | Kruskal-Wallis test | 0.0042 |
| MGC803 | AEG-1 | WT vs S308A | 1±0.1317 ; 1.09±0.0945 | Kruskal-Wallis test | 0.5350 |
|  |  | WT vs S308D | 1±0.1317 ; 1.02±0.154 | Kruskal-Wallis test | 0.9685 |
|  | p-p65 | WT vs S308A | 1±0.1726 ; 1.591±0.0406 | Kruskal-Wallis test | <0.0001 |
|  |  | WT vs S308D | 1±0.1726 ; 0.49±0.0478 | Kruskal-Wallis test | <0.0001 |
|  | p65 | WT vs S308A | 1±0.131 ; 1.003±0.1602 | Kruskal-Wallis test | 0.9994 |
|  |  | WT vs S308D | 1±0.131 ; 1.031±0.1192 | Kruskal-Wallis test | 0.9259 |
|  | eIF4E | WT vs S308A | 1±0.07436 ; 1.578±0.0208 | Kruskal-Wallis test | <0.0001 |
|  |  | WT vs S308D | 1±0.07436 ; 0.4485±0.1083 | Kruskal-Wallis test | <0.0001 |
| MKN45 | AEG-1 | WT vs S308A | 1±0.1356 ; 1.019±0.1156 | Kruskal-Wallis test | 0.9584 |
|  |  | WT vs S308D | 1±0.1356 ; 1.027±0.1203 | Kruskal-Wallis test | 0.9160 |
|  | p-p65 | WT vs S308A | 1±0.1392 ; 1.593±0.06418 | Kruskal-Wallis test | <0.0001 |
|  |  | WT vs S308D | 1±0.1392 ; 0.5752±0.04451 | Kruskal-Wallis test | <0.0001 |
|  | p65 | WT vs S308A | 1±0.07581 ; 0.9544±0.1218 | Kruskal-Wallis test | 0.7827 |
|  |  | WT vs S308D | 1±0.07581 ; 0.9564±0.1117 | Kruskal-Wallis test | 0.7989 |
|  | eIF4E | WT vs S308A | 1±0.03871 ; 1.491±0.04385 | Kruskal-Wallis test | <0.0001 |
|  |  | WT vs S308D | 1±0.03871 ; 0.7327±0.01584 | Kruskal-Wallis test | 0.0040 |

**Figure3 B**

| **Comparsion Group** | **Mean ± SD** | **Statistical Test** | **p-value** |
| --- | --- | --- | --- |
| WT vs Vector | 3.222±0.08063 ; 1±0 | one-way ANOVA | <0.0001 |
| WT vs S308A | 3.222±0.08063 ; 3.904±0.4023 | one-way ANOVA | <0.0001 |
| WT vs S308D | 3.222±0.08063 ; 2.175±0.2742 | one-way ANOVA | 0.0010 |

**Figure3 C**

| **Comparsion Group** | | **Mean ± SD** | **Statistical Test** | **p-value** |
| --- | --- | --- | --- | --- |
| AEG-1 | WT vs S426A | 1 ± 0.037 ; 1.033 ±0.08025 | Kruskal-Wallis test | >0.9999 |
|  | WT vs S426D | 1 ± 0.037 ; 1.049±0.06255 | Kruskal-Wallis test | >0.9999 |
|  | WT vs S308/426A | 1 ± 0.037 ; 1.024 ± 0.06182 | Kruskal-Wallis test | >0.9999 |
|  | WT vs S308/426D | 1 ± 0.037 ; 1.01 ± 0.05819 | Kruskal-Wallis test | >0.9999 |
|  | S426A vs S308/426A | 1.033 ±0.08025 ; 1.024 ± 0.06182 | Kruskal-Wallis test | >0.9999 |
|  | S426D vs S308/426D | 1.049±0.06255 ; 1.01 ± 0.05819 | Kruskal-Wallis test | >0.9999 |
| eIF4E | WT vs S426A | 1 ± 0.0815 ; 1.451 ±0.03891 | Kruskal-Wallis test | <0.0001 |
|  | WT vs S426D | 1 ± 0.0815 ; 0.6347±0.007128 | Kruskal-Wallis test | <0.0001 |
|  | WT vs S308/426A | 1 ± 0.0815 ; 1.989±0.1052 | Kruskal-Wallis test | <0.0001 |
|  | WT vs S308/426D | 1 ± 0.0815 ; 0.2481±0.04133 | Kruskal-Wallis test | <0.0001 |
|  | S426A vs S308/426A | 1.451 ±0.03891 ; 1.989±0.1052 | Kruskal-Wallis test | <0.0001 |
|  | S426D vs S308/426D | 0.6347±0.007128; 0.2481±0.04133 | Kruskal-Wallis test | <0.0001 |

**Figure3 D**

| **Comparsion Group** | | **Mean ± SD** | **Statistical Test** | **p-value** |
| --- | --- | --- | --- | --- |
| AEG-1 | Control vs CXCL12 | 1 ± 0.1889 ; 2.117±0.1796 | Kruskal-Wallis test | <0.0001 |
|  | Control vs TNF-α | 1 ± 0.1889 ; 2.606±0.02102 | Kruskal-Wallis test | <0.0001 |
| p65 | Control vs CXCL12 | 1 ±0.1625 ; 1.141 ±0.1372 | Kruskal-Wallis test | 0.4250 |
|  | Control vs TNF-α | 1 ±0.1625 ; 3.049±0.1332 | Kruskal-Wallis test | <0.0001 |

**Figure3 E**

| **Comparsion Group** | | **Mean ± SD (5 Days)** | **Statistical Test** | **p-value** |
| --- | --- | --- | --- | --- |
| BGC823 | WT vs S308A | 16.93 ± 0.607 ; 20.86 ± 0.675 | one-way ANOVA | <0.0001 |
|  | WT vs S308D | 16.93 ± 0.607 ; 13.08 ± 0.365 | one-way ANOVA | <0.0001 |
| MKN45 | WT vs S308A | 16.93 ± 0.337 ; 18.85 ± 0.92 | one-way ANOVA | <0.0001 |
|  | WT vs S308D | 16.93 ± 0.337 ; 10.08 ± 0.86 | one-way ANOVA | <0.0001 |

**Figure3 F**

| **Comparsion Group** | | **Mean ± SD** | **Statistical Test** | **p-value** |
| --- | --- | --- | --- | --- |
| BGC823 | WT vs S308A | 623 ±32.05 ;721.7 ±34.03 | Kruskal-Wallis test | 0.0480 |
|  | WT vs S308D | 623 ±32.05 ; 510 ±65 | Kruskal-Wallis test | 0.0396 |
| MKN45 | WT vs S308A | 637 ± 31.8 ; 863 ±43.86 | Kruskal-Wallis test | 0.0004 |
|  | WT vs S308D | 637 ± 31.8 ; 415±66.34 | Kruskal-Wallis test | 0.0004 |

**Figure3 G**

| **Comparsion Group** | | **Mean ± SD** | **Statistical Test** | **p-value** |
| --- | --- | --- | --- | --- |
| BGC823 | WT vs S308A | 689.7 ± 106.1 ; 1105.7 ± 120 | Kruskal-Wallis test | 0.0001 |
|  | WT vs S308D | 689.7 ± 106.1 ; 357 ± 29 | Kruskal-Wallis test | 0.0008 |
| MKN45 | WT vs S308A | 658 ± 51.51 ; 858.3 ± 99.17 | Kruskal-Wallis test | 0.0248 |
|  | WT vs S308D | 658 ± 51.51 ; 325 ±65 | Kruskal-Wallis test | 0.0008 |

**Figure4 A**

| **Comparsion Group** | | | **Mean ± SD** | **Statistical Test** | **p-value** |
| --- | --- | --- | --- | --- | --- |
| BGC823 | AEG-1 | WT vs S308/426A | 1 ±0.2105 ; 1.005±0.1539 | one-way ANOVA | 0.9986 |
|  |  | WT vs S308/426D | 1 ±0.2105 ; 0.9796±0.169 | one-way ANOVA | 0.9805 |
|  | p-p65 | WT vs S308/426A | 1 ±0.1189 ; 1.76 ± 0.07253 | one-way ANOVA | <0.0001 |
|  |  | WT vs S308/426D | 1 ±0.1189 ; 0.5463±0.1112 | one-way ANOVA | 0.0035 |
|  | eIF4E | WT vs S308/426A | 1 ±0.1517 ; 1.574 ± 0.1883 | one-way ANOVA | 0.0004 |
|  |  | WT vs S308/426D | 1 ±0.1517 ; 0.3801±0.15 | one-way ANOVA | 0.0002 |
| MGC803 | AEG-1 | WT vs S308/426A | 1 ±0.1648 ; 0.9673±0.1639 | one-way ANOVA | 0.9700 |
|  |  | WT vs S308/426D | 1 ±0.1648 ; 0.9305±0.03652 | one-way ANOVA | 0.8731 |
|  | p-p65 | WT vs S308/426A | 1 ±0.2177 ; 1.802 ±0.2728 | one-way ANOVA | 0.0002 |
|  |  | WT vs S308/426D | 1 ±0.2177 ; 0.5645±0.05808 | one-way ANOVA | 0.0257 |
|  | eIF4E | WT vs S308/426A | 1 ±0.2247 ; 1.779 ±0.3356 | one-way ANOVA | 0.0002 |
|  |  | WT vs S308/426D | 1 ±0.2247 ; 0.4251±0.0381 | one-way ANOVA | 0.0039 |
| MKN45 | AEG-1 | WT vs S308/426A | 1 ±0.229 ; 0.9175 ±0.17 | one-way ANOVA | 0.6706 |
|  |  | WT vs S308/426D | 1 ±0.229 ; 0.9619±0.1762 | one-way ANOVA | 0.9144 |
|  | p-p65 | WT vs S308/426A | 1 ±0.1153 ; 1.543±0.08302 | one-way ANOVA | 0.0002 |
|  |  | WT vs S308/426D | 1 ±0.1153 ; 0.4698±0.07937 | one-way ANOVA | 0.0002 |
|  | eIF4E | WT vs S308/426A | 1 ±0.09852 ; 1.512±0.05217 | one-way ANOVA | 0.0003 |
|  |  | WT vs S308/426D | 1 ±0.09852 ; 0.4226±0.08387 | one-way ANOVA | <0.0001 |

**Figure4 B**

| **Comparsion Group** | | **Mean ± SD** | **Statistical Test** | **p-value** |
| --- | --- | --- | --- | --- |
| BGC823 | WT vs Vector | 6.203 ±0.8832 ; 1 ±0 | Kruskal-Wallis test | <0.0001 |
|  | WT vs S308/426A | 6.203 ±0.8832 ; 16.24±1.28 | Kruskal-Wallis test | <0.0001 |
|  | WT vs S308/426D | 6.203 ±0.8832 ; 2.976±0.3871 | Kruskal-Wallis test | 0.0002 |
| MGC803 | WT vs Vector | 6.362 ±0.7889 ; 1 ±0 | Kruskal-Wallis test | <0.0001 |
|  | WT vs S308/426A | 6.362 ±0.7889 ; 10.84 ±0.7847 | Kruskal-Wallis test | <0.0001 |
|  | WT vs S308/426D | 6.362 ±0.7889 ; 3.429±0.802 | Kruskal-Wallis test | 0.0005 |

**Figure4 C**

| **Comparsion Group** | | **Mean ± SD** | **Statistical Test** | **p-value** |
| --- | --- | --- | --- | --- |
| BGC823 | WT vs Vector | 6.336±1.2 ; 1 ±0 | Kruskal-Wallis test | <0.0001 |
|  | WT vs S308/426A | 6.336±1.2 ; 9.574 ± 0.9196 | Kruskal-Wallis test | <0.0001 |
|  | WT vs S308/426D | 6.336±1.2 ; 2.666±0.4003 | Kruskal-Wallis test | <0.0001 |
| MGC803 | WT vs Vector | 3.722±0.2661 ; 1 ±0 | Kruskal-Wallis test | <0.0001 |
|  | WT vs S308/426A | 3.722±0.2661 ; 7.211±0.2877 | Kruskal-Wallis test | <0.0001 |
|  | WT vs S308/426D | 3.722±0.2661 ; 2.222±0.3017 | Kruskal-Wallis test | 0.0158 |

**Figure4 D**

| **Comparsion Group** | | **Mean ± SD (5 Days)** | **Statistical Test** | **p-value** |
| --- | --- | --- | --- | --- |
| BGC823 | WT vs S308/426A | 14.93±0.39 ; 18.34±0.537 | one-way ANOVA | <0.0001 |
|  | WT vs S308/426D | 14.93±0.39 ; 10.58±1.171 | one-way ANOVA | <0.0001 |
| MGC803 | WT vs S308/426A | 8.757±0.35 ; 12.14±0.442 | one-way ANOVA | <0.0001 |
|  | WT vs S308/426D | 8.757±0.35 ; 6.701±0.192 | one-way ANOVA | <0.0001 |
| MKN45 | WT vs S308/426A | 12.73±1.294 ; 16.56±1.475 | one-way ANOVA | <0.0001 |
|  | WT vs S308/426D | 12.73±1.294 ; 9.374±0.976 | one-way ANOVA | <0.0001 |

**Figure4 E**

| **Comparsion Group** | | **Mean ± SD** | **Statistical Test** | **p-value** |
| --- | --- | --- | --- | --- |
| BGC823 | WT vs S308/426A | 394±50.12 ; 631.7±34.02 | Kruskal-Wallis test | <0.0001 |
|  | WT vs S308/42D | 394±50.12 ; 259±29.51 | Kruskal-Wallis test | 0.0006 |
| MKN45 | WT vs S308/42A | 421.7±25.03 ; 755±30 | Kruskal-Wallis test | <0.0001 |
|  | WT vs S308/42D | 421.7±25.03 ; 216.7±20.03 | Kruskal-Wallis test | <0.0001 |

**Figure4 F**

| **Comparsion Group** | | **Mean ± SD** | **Statistical Test** | **p-value** |
| --- | --- | --- | --- | --- |
| BGC823 | WT vs S308/426A | 691.7±30.01 ； 1139.3±65.61 | Kruskal-Wallis test | <0.0001 |
|  | WT vs S308/42D | 691.7±30.01 ； 313±30 | Kruskal-Wallis test | <0.0001 |
| MKN45 | WT vs S308/42A | 553±45.51 ； 1000.7±119 | Kruskal-Wallis test | <0.0001 |
|  | WT vs S308/42D | 553±45.51 ；390±40 | Kruskal-Wallis test | 0.0151 |

**Figure5 B**

| **Comparsion Group** | | **Mean ± SD (21 Days)** | **Statistical Test** | **p-value** |
| --- | --- | --- | --- | --- |
| BGC823 | WT vs Vector | 982.4±196.9 ；540.9±134.5 | one-way ANOVA | <0.0001 |
|  | WT vs S308/426D | 982.4±196.9 ；651.8±156.9 | one-way ANOVA | 0.0039 |

**Figure5 C**

| **Comparsion Group** | | **Mean ± SD (21 Days)** | **Statistical Test** | **p-value** |
| --- | --- | --- | --- | --- |
| BGC823 | WT vs Vector | 0.9428±0.1395 ; 0.5182±0.1243 | one-way ANOVA | 0.0002 |
|  | WT vs S308/426D | 0.9428±0.1395 ;0.6308±0.09402 | one-way ANOVA | 0.0028 |

**Figure 5 D**

| **Comparsion Group** | | | **Mean ± SD** | **Statistical Test** | **p-value** |
| --- | --- | --- | --- | --- | --- |
| BGC823 | AEG-1 | WT vs Vector | 1.604±0.07535 ; 1±0.09458 | one-way ANOVA | <0.0001 |
|  |  | WT vs S308/426D | 1.604±0.07535;1.927±0.05676 | one-way ANOVA | <0.0001 |
|  | p-p65 | WT vs Vector | 2.097±0.1178 ; 1±0.1129 | one-way ANOVA | <0.0001 |
|  |  | WT vs S308/426D | 2.097±0.1178;1.368±0.1004 | one-way ANOVA | <0.0001 |
|  | eIF4E | WT vs Vector | 2.673±0.1525 ; 1±0.132 | one-way ANOVA | <0.0001 |
|  |  | WT vs S308/426D | 2.673±0.1525 ; 1.506±0.06777 | one-way ANOVA | <0.0001 |

**Figure 5 E**

| **Comparsion Group** | | | **Mean ± SD** | **Statistical Test** | **p-value** |
| --- | --- | --- | --- | --- | --- |
| BGC823 | *eIF4E* | WT vs Vector | 5.2±0.94 ; 1±0 | one-way ANOVA | <0.0001 |
|  |  | WT vs S308/426D | 5.2±0.94 ; 1.828±0.4126 | one-way ANOVA | <0.0001 |
|  | *FOS* | WT vs Vector | 4.62±0.7993 ; 1±0 | one-way ANOVA | <0.0001 |
|  |  | WT vs S308/426D | 4.62±0.7993 ; 2.128±0.7509 | one-way ANOVA | <0.0001 |
|  | *IL8* | WT vs Vector | 6.873±0.6975 ; 1±0 | one-way ANOVA | <0.0001 |
|  |  | WT vs S308/426D | 6.873±0.6975 ; 2.65±0.385 | one-way ANOVA | <0.0001 |

**Figure5 F**

| **Comparsion Group** | | **Mean ± SD** | **Statistical Test** | **p-value** |
| --- | --- | --- | --- | --- |
| BGC823 | WT vs Vector | 60±8.832 ; 34.17±8.886 | one-way ANOVA | 0.0001 |
|  | WT vs S308/426D | 60±8.832 ; 36.33±6.532 | one-way ANOVA | 0.0003 |

**Figure5 G**

| **Comparsion Group** | | **Mean ± SD** | **Statistical Test** | **p-value** |
| --- | --- | --- | --- | --- |
| BGC823 | WT vs Vector | 43.33±9.564 ; 17.67±4.719 | one-way ANOVA | <0.0001 |
|  | WT vs S308/426D | 43.33±9.564 ; 24.718±4.131 | one-way ANOVA | =0.0003 |

**Figure 7 A**

| **Comparsion Group** | | **Mean ± SD** | **Statistical Test** | **p-value** |
| --- | --- | --- | --- | --- |
| PPP1R21 | PPP1R21 vs Vector | 6.157±1.197 ; 1±0.09791 | Kruskal-Wallis test | <0.0001 |
| p-p65 | PPP1R21 vs Vector | 2.144±0.274 ; 1±0.07012 | Kruskal-Wallis test | 0.0275 |
| eIF4E | PPP1R21 vs Vector | 2.179±0.3021 ; 1±0.09521 | Kruskal-Wallis test | 0.0226 |
| P-AEG-1(S426) | PPP1R21 vs Vector | 0.3163±0.0939 ; 1±0.08215 | Kruskal-Wallis test | 0.0292 |

**Figure 7 B**

| **Comparsion Group** | **Mean ± SD(5 Days)** | **Statistical Test** | **p-value** |
| --- | --- | --- | --- |
| PPP1R21 vs Vector | 14.23±1.23 ; 8.024±1.029 | one-way ANOVA | <0.0001 |

**Figure 7 C**

| **Group** | **Mean ± SD** | **Statistical Test** | **p-value** |
| --- | --- | --- | --- |
| Colony | 952±40 ; 400.7±22.14 | Unpaired t test | P<0.0001 |
| Transwell | 132±13.53 ; 69.67±17.79 | Unpaired t test | P=0.0085 |

**Figure 7 D**

| **Comparsion Group** | | **Mean ± SD** | **Statistical Test** | **p-value** |
| --- | --- | --- | --- | --- |
| PPP1R21 | PPP1R21 siRNA vs Control | 0.4134±0.01578 ; 1±0.02561 | Kruskal-Wallis test | P<0.0001 |
| p-p65 | PPP1R21 siRNA vs Control | 0.3219±0.03511 ; 1±0.0572 | Kruskal-Wallis test | P<0.0001 |
| eIF4E | PPP1R21 siRNA vs Control | 0.4699±0.02662 ; 1±0.0871 | Kruskal-Wallis test | P<0.0001 |
| P-AEG-1(S426) | PPP1R21 siRNA vs Control | 1.714±0.1242 ; 1±0.08797 | Kruskal-Wallis test | P<0.0001 |

**Figure 7 E**

| **Comparsion Group** | **Mean ± SD(5 Days)** | **Statistical Test** | **p-value** |
| --- | --- | --- | --- |
| PPP1R21 siRNA vs Control | 4.201±0.5489 ; 11.02±1.229 | Kruskal-Wallis test | P<0.0001 |

**Figure 7 F**

| **Group** | **Mean ± SD** | **Statistical Test** | **p-value** |
| --- | --- | --- | --- |
| Colony | 226.3±22.74 ; 411±21.52 | Unpaired t test | P=0.0005 |
| Transwell | 25±6 ; 78.67±17.16 | Unpaired t test | P=0.0069 |

**Supplementary fig. S3A**

| **Comparsion Group** | | | **Mean ± SD** | **Statistical Test** | **p-value** |
| --- | --- | --- | --- | --- | --- |
| BGC823 | AEG-1 | WT vs Vector | 1.582±0.04808 ; 1±0.05457 | one-way ANOVA | <0.0001 |
|  |  | WT vs S308/426D | 1.582±0.04808 ; 1.687±0.06616 | one-way ANOVA | 0.0866 |
|  | p-p65 | WT vs Vector | 2±0.006356 ; 1±0.04558 | one-way ANOVA | <0.0001 |
|  |  | WT vs S308/426D | 2±0.006356 ; 1.453±0.08172 | one-way ANOVA | <0.0001 |
|  | eIF4E | WT vs Vector | 2.135±0.08596 ; 1±0.05388 | one-way ANOVA | <0.0001 |
|  |  | WT vs S308/426D | 2.135±0.08596 ; 1.441±0.06306 | one-way ANOVA | <0.0001 |

**Supplementary fig. S3B**

| **Comparsion Group** | | **Mean ± SD(5 Days)** | **Statistical Test** | **p-value** |
| --- | --- | --- | --- | --- |
| BGC823 | WT vs Vector | 16.07±0.686 ; 10.41±0.547 | one-way ANOVA | <0.0001 |
|  | WT vs S308/426D | 16.07±0.686 ; 13.895±0.648 | one-way ANOVA | <0.0001 |

**Supplementary fig. S3C**

| **Comparsion Group** | | **Mean ± SD** | **Statistical Test** | **p-value** |
| --- | --- | --- | --- | --- |
| BGC823 | WT vs Vector | 858±55.51 ; 573±32.05 | one-way ANOVA | 0.0002 |
|  | WT vs S308/426D | 858±55.51 ; 369±9.849 | one-way ANOVA | <0.0001 |

**Supplementary fig. S3D**

| **Comparsion Group** | | **Mean ± SD** | **Statistical Test** | **p-value** |
| --- | --- | --- | --- | --- |
| BGC823 | WT vs Vector | 1252±255.5 ; 414±97.53 | one-way ANOVA | 0.0030 |
|  | WT vs S308/426D | 1252±255.5 ; 607±185 | one-way ANOVA | 0.0108 |

**Supplementary fig. S5C**

| **Comparsion Group** | | | **Mean ± SD** | **Statistical Test** | **p-value** |
| --- | --- | --- | --- | --- | --- |
| BGC823 | AEG-1 | WT vs Vector | 3.101±0.1458 ; 1±0.08705 | Kruskal-Wallis test | <0.0001 |
|  |  | WT vs S308A | 3.101±0.1458 ; 3.088±0.2389 | Kruskal-Wallis test | 0.9995 |
|  |  | WT vs S308D | 3.101±0.1458 ; 3.043±0.274 | Kruskal-Wallis test | 0.9546 |
|  | p-AEG-1(S426) | WT vs Vector | 3.295±0.2588 ; 1±0.07131 | Kruskal-Wallis test | <0.0001 |
|  |  | WT vs S308A | 3.295±0.2588 ; 3.382±0.03618 | Kruskal-Wallis test | 0.8688 |
|  |  | WT vs S308D | 3.295±0.2588 ; 3.241±0.02988 | Kruskal-Wallis test | 0.9617 |

**Supplementary fig. S5D**

| **Comparsion Group** | | | **Mean ± SD** | **Statistical Test** | **p-value** |
| --- | --- | --- | --- | --- | --- |
| BGC823 | Flag | WT vs Vector | 5.594±0.2499 ; 1±0.07743 | one-way ANOVA | <0.0001 |
|  |  | WT vs S308A | 5.594±0.2499 ; 5.556±0.2901 | one-way ANOVA | 0.9984 |
|  |  | WT vs S308D | 5.594±0.2499 ; 5.453±0.1072 | one-way ANOVA | 0.9323 |
|  | AEG-1 | WT vs Vector | 5.109±0.04968 ; 1±0.0708 | one-way ANOVA | <0.0001 |
|  |  | WT vs S308A | 5.109±0.04968 ; 5.138±0.1934 | one-way ANOVA | 0.9993 |
|  |  | WT vs S308D | 5.109±0.04968 ; 5.164±0.1175 | one-way ANOVA | 0.9954 |
|  | p-AEG-1(S426) | WT vs Vector | 2.581±0.307 ; 1±0.03839 | one-way ANOVA | <0.0001 |
|  |  | WT vs S308A | 2.581±0.307 ; 1.672±0.2399 | one-way ANOVA | 0.0134 |
|  |  | WT vs S308D | 2.581±0.307 ; 6.413±1.086 | one-way ANOVA | <0.0001 |
